# Immunotherapy With 5, 15-DPP Mediates Macrophage M1 Polarization and Modulates Subsequent *Mycobacterium tuberculosis* Infectivity in rBCG30 Immunized Mice

**DOI:** 10.3389/fimmu.2021.706727

**Published:** 2021-10-29

**Authors:** Faraz Ahmad, Mohd. Saad Umar, Nazoora Khan, Fauzia Jamal, Pushpa Gupta, Swaleha Zubair, Umesh Datta Gupta, Mohammad Owais

**Affiliations:** ^1^ Molecular Immunology Lab, Interdisciplinary Biotechnology Unit, Aligarh Muslim University, Aligarh, India; ^2^ Bio-Safety Level (BSL)-3 Animal Experimentation Facility, Indian Council of Medical Research (ICMR)-National Japanese Leprosy Mission for Asia (JALMA) Institute for Leprosy and Other Mycobacterial Diseases, Agra, India; ^3^ Department of Computer Science, Aligarh Muslim University, Aligarh, India

**Keywords:** tuberculosis, vaccine, immunotherapeutics, IL-10 (interleukin 10), 5, 15-diphenylporphyrin

## Abstract

Tuberculosis (TB) is a significant and continuing problem worldwide, with a death toll of around 1.5 million human lives annually. BCG, the only vaccine against TB, offers a varied degree of protection among human subjects in different regions and races of the world. The majority of the population living near the tropics carries a varying degree of tolerance against BCG due to the widespread prevalence of non-tuberculous mycobacteria (NTM). Interestingly, ≈90% of the *Mycobacterium tuberculosis (Mtb)* infected population restrain the bacilli on its own, which strengthens the notion of empowering the host immune system to advance the protective efficacy of existing mycobacterial vaccines. In general, *Mtb* modulates IL-10/STAT3 signaling to skew host mononuclear phagocytes toward an alternatively activated, anti-inflammatory state that helps it thrive against hostile immune advances. We hypothesized that modulating the IL-10/STAT3 driven anti-inflammatory effects in mononuclear cells may improve the prophylactic ability of TB vaccines. This study investigated the immunotherapeutic ability of a porphyrin based small molecule inhibitor of IL-10/STAT3 axis, 5, 15-diphenyl porphyrin (DPP), in improving anti-TB immunity offered by second generation recombinant BCG30 (rBCG30-ARMF-II^®^) vaccine in mice. The DPP therapy potentiated vaccine induced anti-TB immunity by down-modulating anti-inflammatory responses, while simultaneously up-regulating pro-inflammatory immune effector responses in the immunized host. The employed DPP based immunotherapy led to the predominant activation/proliferation of pro-inflammatory monocytes/macrophages/DCs, the concerted expansion of CD4+/CD8+ effector and central memory T cells, alongside balanced Th17 and Treg cell amplification, and conferred augmented resistance to aerosol *Mtb* challenge in rBCG30 immunized BALB/c mice.

## Introduction

As a strategy to counter immune onslaught, *Mycobacterium tuberculosis (Mtb)*, the causative agent of human tuberculosis (TB), evokes anti-inflammatory responses in the host (1–6). It is tempting to speculate that the down-modulation of anti-inflammatory machinery with simultaneous mobilization of pro-inflammatory effectors, may serve as a deliberate host approach to control *Mtb* infection. Several recent studies have established that mononuclear phagocytes are crucial for imparting protection against *Mtb* infection (7–10). Interestingly, they also possess T cell-like memory capacity against re-infection (9, 11, 12). Along this line, the relatively modest potency of TB vaccines developed to date suggests that most of the T-cell targeting candidate TB vaccines do not contribute to any significant advancement in anti-TB prophylaxis programs. This and the above-specified observations, in turn, indicate the need to develop effective prophylactic strategies that can simultaneously activate both innate and adaptive arms of the immune system.

As a crucial component of first-line immune defense, mononuclear phagocytes, especially macrophages, encounter *Mtb* early during an invasion. It is well established that mononuclear phagocytes restrict the replication of invading *Mtb* long before the involvement and participation of specialized T cells (13). The macrophage subpopulation displaying pro-inflammatory classical or M1 phenotypes play a crucial role in the efficient clearance of invading *Mtb* (14). To withstand the immune onslaught, *Mtb* subverts inflicted macrophages to switch toward an anti-inflammatory M2 phenotype (6, 14–16). However, prolonged inflammation in chronic granulomatous infections (*cf. Schistosoma mansonii)* (17) is detrimental and triggers immunopathological consequences in the host. In general exuberant innate inflammation is associated with poor resolution of TB (18). Thus, a well-coordinated and temporally balanced expansion of both pro- and anti-inflammatory monocytes/macrophages is essential for achieving optimal protection against *Mtb*.

The role of IL-10 in exacerbating anti-mycobacterial immunity is well established (19–21) and has been reviewed elsewhere (22). The administration of IL-10 neutralizing monoclonal antibody during BCG vaccination ameliorated protection against subsequent *Mtb* challenge, in both- susceptible and resistant mice strains (23). Furthermore, immunization of IL-10 knockout mice with BCG resulted in elevated anti-mycobacterial immunity upon challenge with *Mtb* (24). Considering these facts, it is tempting to explore strategies that can simultaneously reinforce both the innate and adaptive arms of the immune system as a means to achieve desirable prophylaxis against *Mtb.* On this line, IL-10 mediated activation of JAK-STAT pathway and subsequent STAT3 activity is well established (5, 25, 26). STAT3 is a long established therapeutic target in cancer (27, 28). IL-10 driven STAT3 signaling in macrophages has been associated with the development of ocular angiogenesis and macular degeneration (26). Administration of the small molecule, 5,15-di phenyl porphyrin (DPP), a selective inhibitor of IL-10/STAT3 signaling, has been shown to reduce pathologic neovascularization and Age-related Macular Degeneration (AMD) *via* inhibition of alternative activation and M2 polarization of macrophages (26). DPP is a small molecular weight organic compound containing a central porphyrin ring similar to phytochrome chlorophyl or heme, an integral component of hemoglobin. Due to its natural origin, it is a relatively safe and attractive STAT3 inhibitor over many other synthetic STAT3 antagonists. The role of the IL-10/STAT3 signaling axis has also been discussed recently in multiple reports, implicating this cascade in modulating monocytes/macrophages toward an anti-inflammatory, alternatively activated state conducive for *Mtb* outgrowth (3, 6, 29–33).

Therefore, targeting and transiently disrupting the IL-10/STAT3 axis mediated immune-suppressive effects could be effective. This study tested this approach to improve the anti-TB efficacy of the second-generation rBCG30-ARMF-II^®^ vaccine. The vaccine is devoid of antibiotic resistance marker and expresses 2.6 fold more Ag85B than the original construct (34), which was shown to be safe and immunogenic in a phase 1 clinical trial (35). The rBCG30-ARMF-II^®^ vaccine provided improved protection against *Mtb* aerosol challenge than classical BCG in guinea pigs (34). It also induced a strong, cross-protective immune response against *M. leprae* antigens in mice (36).

In the present study, we attempted to bolster rBCG30 mediated anti-TB immunity by modulating host IL-10/STAT3 signaling orchestrated anti-inflammatory effects. We administered DPP, a small molecule inhibitor of the IL-10/STAT3 signaling (26, 37), in mice at the post- rBCG30 vaccination stage (designated as Post Vaccination Immunotherapy or PVI). In an alternative approach, DPP was administered post challenge with *Mtb* in already vaccinated (with rBCG30) animals (herein called Post Infection Immunotherapy or PII). The treatment of mice with DPP resulted in a reduced expansion of pathogen permissive AAMs (Alternatively Activated Monocytes/Macrophages) along with the reciprocal predominance of pro-inflammatory CAMs (Classically Activated Monocytes/Macrophages), the cells that resist the establishment of successful *Mtb* infection. The results of the present study suggest that the modulation of the IL-10/STAT3 axis mediated anti-inflammatory effects can be a viable new anti-TB strategy, especially in vaccinated hosts.

## Results

### Immunotherapy With DPP Skews Mononuclear Phagocytes Toward a Pro-Inflammatory CAM State

**Figure 1:**
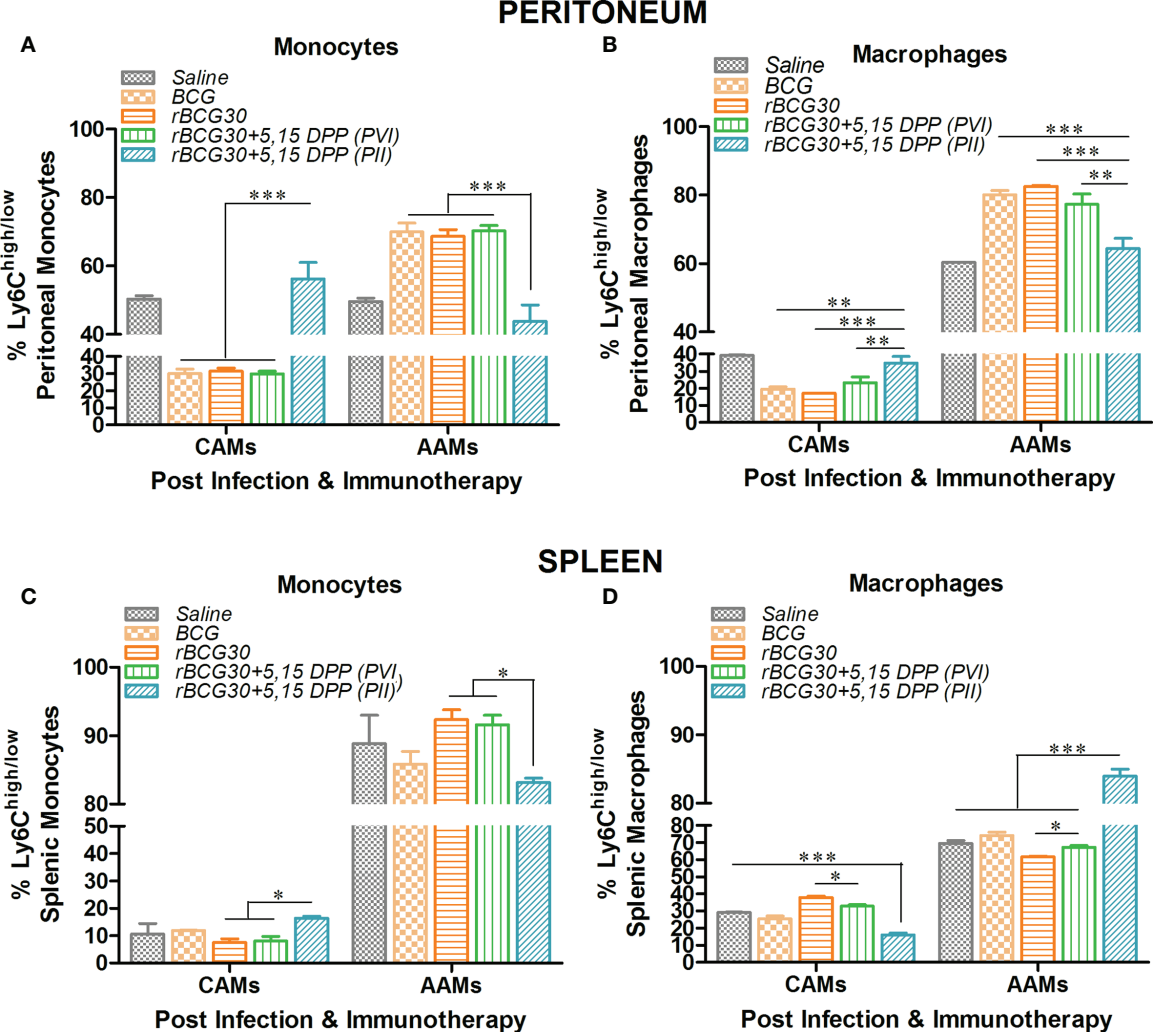
The magnitude and phenotype of peritoneal and splenic monocyte/macrophage populations at 2 weeks post-challenge. Bar graphs in the figure represent percent numbers of Ly6C^high^ CAMs and Ly6C^low^ AAMs among **(A, C)** CD11b^+^F4/80^-^SSC^low^ monocytes, and **(B, D)** CD11b^+^F4/80^+^SSC^low^ macrophages. The cells were isolated from either peritoneum (upper panel) or spleen (lower panel) of the mice (n=3) belonging to various experimental groups the next day following completion of post-infection immunotherapy (PII) schedule (2 weeks PC with *Mtb)* and immunophenotyped employing flow cytometry. The results depicted in the figure are representative of two independent experiments and are presented as means ± SEM of one of the two experiments with similar observations. The statistical significance of the difference between various groups was performed by employing two-way ANOVA followed by Bonferroni’s multiple comparison post-test. The *p* values, <0.05(*), <0.01(**), <0.001(***) were considered as significant for analysis and interpretation of experimental data.

Ly6C+ mononuclear phagocytes are largely divided into two subpopulations-Ly6C^hi^ and Ly6C^low^, which are further defined as pro-inflammatory M1 or CAMs/CADCs and anti-inflammatory M2 or AAMs/AADCs, respectively (38–42). There is a direct correlation between expression of Ly6C and the degree of the functional differentiation state of mononuclear phagocytes following infection with *Mtb* (42, 43), and other intracellular pathogens (17, 40). Considering this, we intended to restrict the *Mtb* induced polarization of monocytes/macrophages/DCs toward permissive AAMs/AADCs. We treated rBCG30-immunized animals with DPP, either before (PVI) or after (PII) challenge with *Mtb*, to transiently block IL-10/STAT3 mediated anti-inflammatory signaling. Transient blockade of IL-10/STAT3 signaling was used as a means to allow augmented differentiation of mononuclear phagocytes into pathogen-clearing CAMs/CADCs. Interestingly, immunotherapy with DPP in rBCG30-immunized animals (PVI) resulted in significant up-regulation of CAM phenotype in monocytes/macrophages, while fewer monocytes/macrophages with AAM phenotype were present among both peritoneal exudates cells (PEC) population (**Figure S1**) and splenic mononuclear cells (**Figure S2**). DPP immunotherapy in rBCG30 vaccinated animals post-challenge with *Mtb* (PII) also resulted in significant augmentation of the CAMs among monocyte/macrophage population from peritoneal exudates (**Figures 1A, B**). Although in the spleen, only monocytes were significantly expanded into CAMs, while macrophages were predominantly AAMs, when compared with either controls or other experimental groups (**Figures 1C, D**).

**Figure 2:**
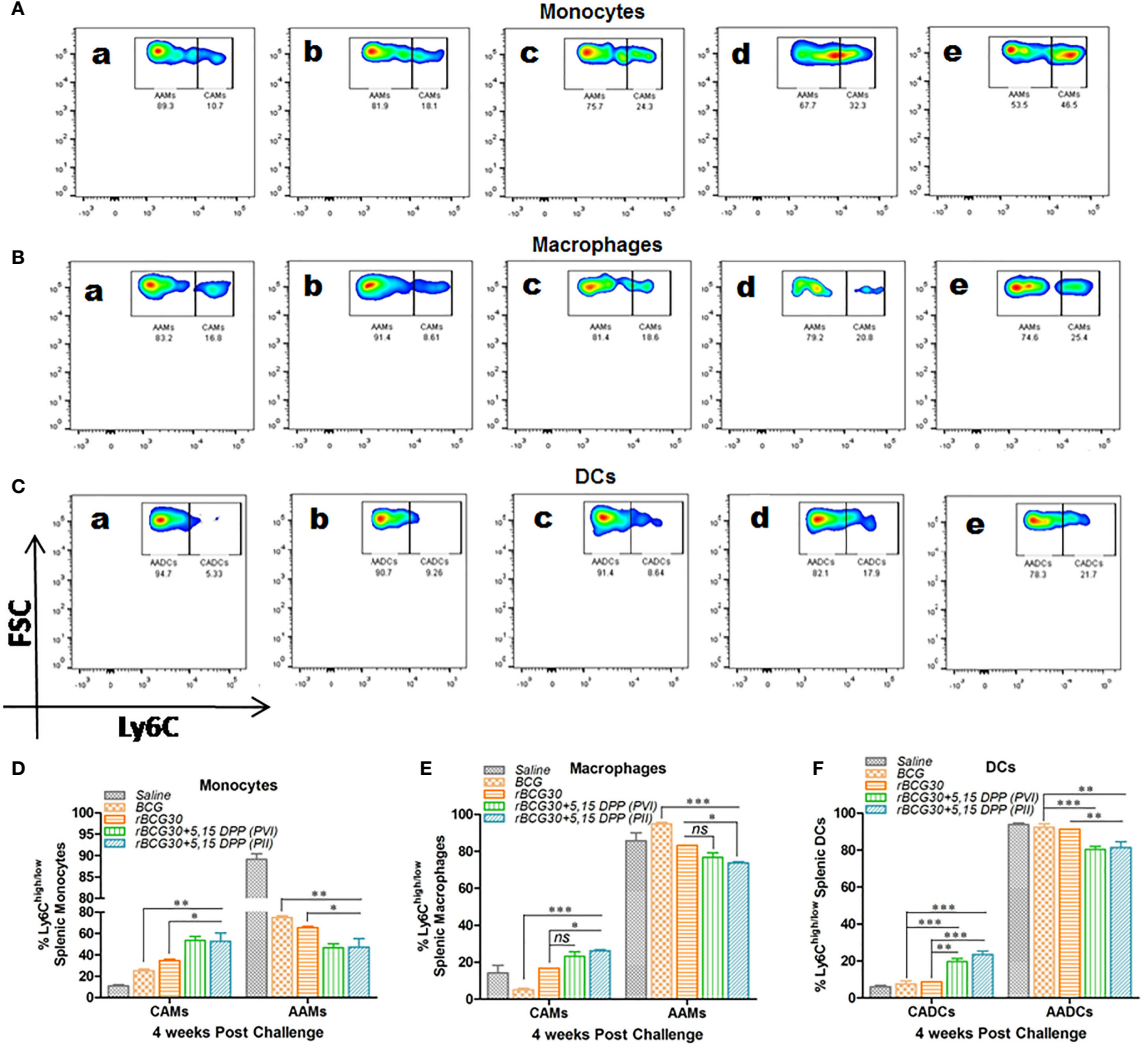
Comparative abundance of the classical and alternative phenotype bearing splenic monocytes/macrophages/DCs at week 4 post-challenge. Representative FACS micrographs **(A–C)** and corresponding bar graphs **(D–F)** in the figure represent the percent frequency of Ly6C^high^ CAMs/CADCs and Ly6C^low^ AAMs/AADCs among **(A, D)** CD11b^+^F4/80^-^SSC^low^monocytes, **(B, E)** CD11b^+^F4/80^+^SSC^low^ macrophages, and **(C, F)** CD11b^-^F4/80^-^SSC^low^ DCs isolated from the spleen of mice belonging to various groups and profiled using flow cytometry. The groups included in the study and depicted in the FACS micrographs were: (a) Saline, (b) BCG, (c) rBCG30, (d) rBCG30+5,15-DPP (PVI), and (e) rBCG30+5,15-DPP (PII). The results depicted in the figure are representative of two independent experiments and are presented as means ± SEM of one of the two experiments with similar observations. The significance testing of differences between various groups was performed employing two-way ANOVA followed by Bonferroni’s multiple comparison post-test. The *p* values, <0.05(*), <0.01(**), <0.001(***) were considered as significant for analysis and interpretation of data. ns, non-significant.

The relative abundance of splenic CAMs/CADCs and AAMs/AADCs was assessed at 4- and 8-weeks PC 4- and 8-weeks PC as well. At 4 week PC, rBCG30 immunized animals (administered with either PVI or PII) displayed augmented expansion of both monocytes and DCs with pathogen restricting classical phenotype (CAMs/CADCs). While there was a concomitant reduction in the frequency of the pathogen permissive cells bearing alternative phenotype (AAMs/AADCs), as compared to either untreated, BCG- or rBCG30-immunized control groups. In contrast, macrophages from the PII, but not the PVI group, displayed a similar rise in the expansion of CAMs with a relatively reduced expansion of AAMs, when compared with the rBCG30 control animals (**Figure 2**). This increased expansion of CAMs among splenic macrophages from the PII group indicates the systemic induction of M1 polarized environment in infected macrophages, possibly as a direct effect of DPP therapy.

**Figure 3:**
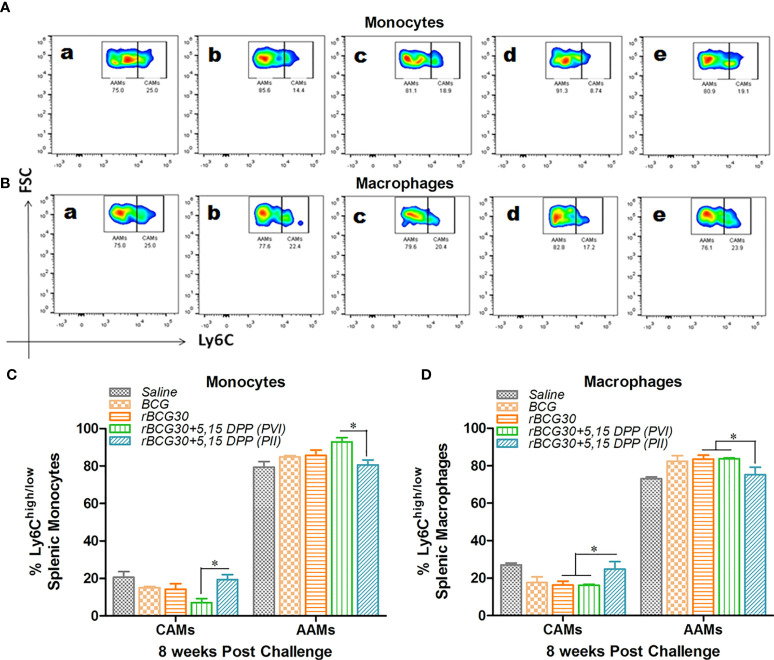
Comparative abundance of classical (CAMs) and alternative (AAMs) splenic monocytes/macrophages at week 8 post-challenge. Representative FACS micrographs and bar graphs in the figure represents the percent frequency of Ly6C^high^ CAMs and Ly6C^low^ AAMs among **(A, C)** CD11b^+^F4/80^-^SSC^low^ monocytes and **(B, D)** CD11b^+^F4/80^+^SSC^low^ macrophages isolated from the spleen of mice (n=4-5) belonging to various groups and profiled using flow cytometry. The various groups included in the study and depicted in the FACS micrographs were: (a) Saline, (b) BCG, (c) rBCG30, (d) rBCG30+5,15-DPP (PVI), and (e) rBCG30+5,15-DPP (PII). The results depicted in the figure are representative of two independent experiments and are presented as means ± SEM of one of the two experiments with similar observations. The significance testing of differences between various groups was performed employing Two-way ANOVA followed by Bonferroni’s multiple comparison post-test. The *p* values, <0.05(*), <0.01(**), <0.001(***) were considered as significant for analysis and interpretation of the data.

Some pathogens, such as *Mtb* (6) and *Brucella* (40) adapt intracellular parasitism and sought shelter in pathogen permissive macrophages of the host. Upon activation, the IL-10/STAT3 axis renders monocytes/macrophages toward an alternative state of activation during *Mtb* infection (6). This generally ensues in the establishment of chronic infection in the host. Taking this into account, we probed the effect of IL-10/STAT3 inhibiting immunotherapy in the modulation of the AAM population during the chronic phase of *Mtb* infection. At week 8 PC, the abundance of CAMs was still significantly higher in the rBCG30+PII group, as compared to the rBCG30+PVI group (**p<0.05,** **Figure 3**). In addition, the administration of DPP (PII) was successful at restricting the expansion of pathogen permissive AAMs (macrophages) in the rBCG30+PII group even during the late phase of infection, as compared to either rBCG30+PVI or rBCG30 alone groups (p<0.05, **Figure 3D**).

### DPP Therapy Induces Pro-Inflammatory Cytokines and Dampens the Production of Key Anti-Inflammatory Cytokines

**Figure 4:**
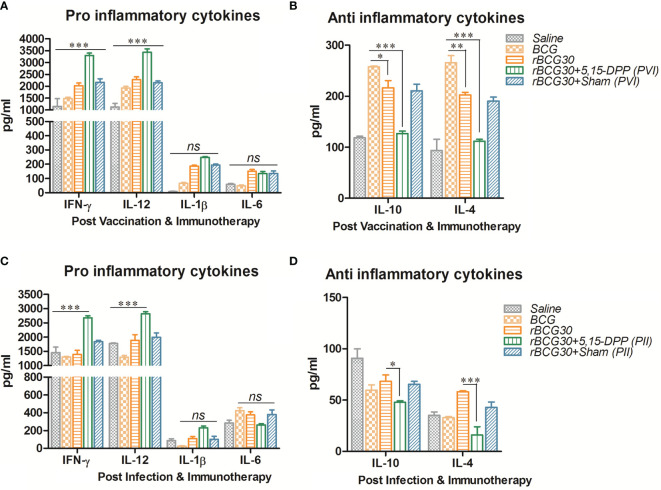
Splenocytes cytokine expression at post vaccination and immunotherapy (PVI) as well as post infection and immunotherapy (PII). The level of signature innate cytokines (IL-1β and IL-6); Th1 cytokines (IFN-γ and IL-12), and anti-inflammatory cytokines (IL-10 and IL-4), was estimated following **(A, B)** PVI and **(C, D)** PII, using sandwich ELISA in the splenocyte culture supernatants belonging to the various immunized groups. Cells were uniformly stimulated with native Ag85B antigen from *Mtb* (5 µg/ml). Differential expression levels of cytokines in various immunized groups are presented as bar diagrams. The data were analyzed by employing Two-way ANOVA followed by Bonferroni’s multiple comparison test and are shown as the means ( ± SEM) of one of the two experiments with similar observations performed in triplicate from the pooled cells of at least three animals per group, where *p* values; *viz.* p<0.05(*), p<0.01(**), and p<0.001(***) were considered significant. ns, non-significant.

Splenocytes from immunized animals were harvested on day 1 post completion of immunotherapy (either PVI or PII), and cultured *ex vivo* as specified in the method section. Signature anti-inflammatory cytokines IL-10 and IL-4, and pro-inflammatory cytokines IFN-γ, IL-12, IL-1β, and IL-6, were assessed in the culture supernatant employing sandwich ELISA. The level of Th1 specific pro-inflammatory cytokines, IFN-γ and IL-12, was found to be elevated in animals that received immunotherapy with DPP post vaccination with rBCG30 (PVI). On the other hand, the level of innate pro-inflammatory effectors, either IL-6 or IL-1β, was not significantly up-regulated, as compared to controls (**Figure 4A**). However, the employed immunotherapy resulted in diminutive expression of IL-10 and IL-4 cytokines in the culture supernatant. The level of these two cytokines was found to be significantly reduced following DPP treatment in the PVI group (**Figure 4B**).

The **s**plenocytes obtained from the group of animals vaccinated with rBCG30 and administered DPP for two weeks following *Mtb* challenge (PII scheme) were also assessed for their potential to produce both pro- (IFN-γ, IL-12, IL-1β, and IL-6) as well as anti- (IL-10 and IL-4) inflammatory cytokines *ex vivo*. Splenic cells from rBCG30 vaccinated animals (belonging to the PII group) displayed impressive production of Th1 cytokines, IFN-γ, and IL-12 (p<0.001, **Figure 4C**). We observed a significant reduction in the level of anti-inflammatory cytokines IL-10 (p<0.05) and IL-4 (p<0.001) in the same group of animals, as compared to rBCG30-immunized controls (**Figure 4D**). Additionally, at 2 weeks PC, the level of either IL-1β or IL-6 was un-influenced by post-infection immunotherapy, when compared to controls (**Figure 4C**).

### DPP Therapy Up-Regulates Pro-Inflammatory Cytokines Until 8 Weeks Post-Challenge in the Immunized Animals

**Figure 5:**
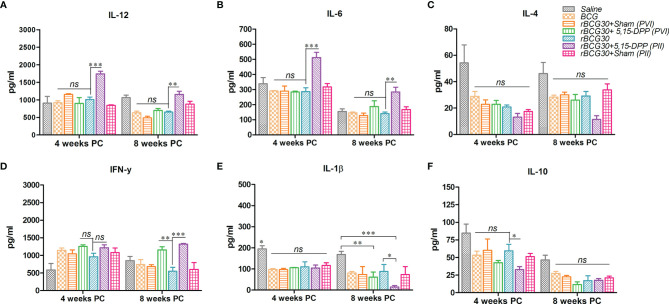
Splenocytes production of signature pro-and anti-inflammatory cytokines at 4 and 8 weeks post-challenge. The level of signature Th1 cytokines IL-12, IFN-γ **(A, D)**, innate cytokines IL-6, IL-1β **(B, E)**, and anti-inflammatory cytokines IL-4, IL-10 **(C, F)**, were estimated using sandwich ELISA in splenocyte culture supernatants belonging to the various groups. Cells were uniformly stimulated with native Ag85B antigen from *Mtb* (5 µg/ml). Differential levels of cytokines between various groups are presented as bar diagrams. The data were analyzed employing Two-way ANOVA followed by Bonferroni’s multiple comparison test and are shown as the means ( ± SEM) from one of the two independent experiments with similar observations deduced from pooled cells of at least three mice per group and performed in technical triplicate, where p<0.05(*), p<0.01(**), and p<0.001(***) were considered significant.

Splenocytes from various groups of immunized animals were assessed for their potential to produce signature pro- and anti-inflammatory cytokines at 4- and 8-weeks PC. The level of IL-12, the master cytokine of the Th1 cell-mediated immunity, was found to be significantly elevated in the rBCG30+PII group both at 4 **(p<0.001)** and 8 **(p<0.01)** weeks PC (**Figure 5A**). The expression of IFN-γ, another important effector cytokine of the Th1 type immunity, was also significantly up-regulated at week 8 PC in the PII group, as compared to rBCG30 alone (p<0.001). There was profuse induction of IFN-γ in the PVI group as well when compared with the rBCG30 alone group (p<0.01) (**Figure 5D**).

We then assessed another crucial pro-inflammatory cytokine Il-6 in various groups of immunized animals. The cytokine IL-6, besides being a crucial effector of innate immune response in TB (12), is also critical for amplification of Th17 cells responses in *Mtb* infected animals (44). Contrary to the 2 week PC time point (**Figure 4C**), the level of IL-6 was up-regulated significantly in rBCG30-immunized animals belonging to the PII group at both 4 (p<0.001) and 8 (p<0.01) weeks PC time points (**Figure 5B**). The level of IL-6 was almost identical in rBCG30+PVI, BCG and saline administered groups of animals at both 4 and 8 weeks PC time points.

IL-1β, an important innate cytokine, is primarily a product of either NLRP3 or AIM2 inflammasome assembly and is reported to play a crucial role in regulating the outcome of *Mtb* infection (18, 45). The level of IL-1β was not significantly influenced by either of the immunotherapeutic strategies employed, until up to 4 weeks PC. Instead, the IL-1β level was found to be increased in the saline (*Mtb* infection control) group, as compared with all other experimental or control groups (p<0.05) (**Figure 5E**). On the other hand, there was a considerable decrease in IL-1β levels in both the rBCG30+PVI **(p<0.01)** and rBCG30+PII (p<0.001) groups at week 8 PC (**Figure 5E**).

The level of signature anti-inflammatory cytokines, IL-10 and IL-4, was probed at stipulated time points post *Mtb* challenge. At 2 weeks PC, the level of both IL-10 and IL-4 was significantly reduced in the rBCG30+PII group (**Figure 4D**). In contrast, at later time points (4 and 8 weeks PC), differences waned, especially the level of IL-4 (**Figure 5C**). However, the level of IL-10, the main regulator of anti-mycobacterial immunity, remained low at 4 weeks PC in animals vaccinated with rBCG30 and treated with DPP following *Mtb* challenge (PII) (**Figure 5F**).

### Post-Infection Immunotherapy With DPP Strengthened the Expansion of CD4+ Multifunctional T Cells

**Figure 6:**
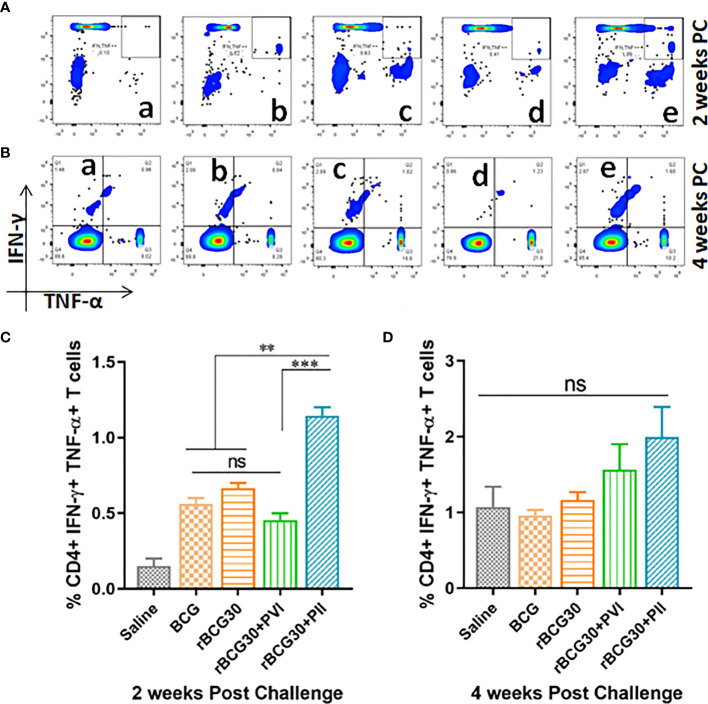
Multifunctional CD4+ T cell response post-challenge with *Mtb*. Splenocytes isolated from various immunized and immunotherapy treated animals were profiled employing flow cytometry for simultaneous production of IFN-γ and TNF-α by CD3+CD4+ T cells at 2 as well as 4 weeks PC. The figure depicts representative FACS plots of CD4+ MFTs belonging to **(a)** Saline, **(b)** BCG, **(c)** rBCG30, **(d)** rBCG30+5,15-DPP (PVI), and **(e)** rBCG30+5,15-DPP (PII) groups, at **(A)** 2 and **(B)** 4 weeks PC, respectively, and corresponding quantitative assessment in the form of bar graphs **(C, D)**. Cells were stimulated with native Ag85B antigen from *Mtb* (5 µg/ml). The data were analyzed using One-way ANOVA followed by Tukey’s multiple comparison test and are shown as the means ( ± SEM) from one of the two independent experiments with similar observations performed in at least three biological replicates, where p<0.05(*), p<0.01(**), and p<0.001(***) were considered significant. ns, non-significant.

Induction of functionally superior multifunctional T cells (MFTs) following immunization and/or infection is an important feature of T cell mediated immunity. Multifunctional T cells are known as important anti-pathogen effectors that are crucial for immunity and protection against intracellular infectious agents including *Mtb* (46–50). The abundance of antigen specific CD4+ T cells induced in various immunized and immunotherapy groups were assessed *ex vivo* for simultaneous expression of Th1 effector cytokines (IFN-γ and TNF-α) at 2 as well as 4 weeks PC (**Figure 6**). Interestingly, rBCG30-immunized animals treated with DPP following *Mtb* challenge (PII scheme) produced significantly more CD4+ MFTs, as compared with rBCG30 alone (p<0.05) or rBCG30+PVI group (p<0.01) at 2 weeks PC. In contrast, PVI treated animals did not induce any better level of CD4+ MFTs either at 2 or 4 weeks PC, as compared to rBCG30 controls (p>0.05) (**Figures 6A, C**). The frequency of CD4+ MFTs induced in saline, BCG, and rBCG30+PVI administered animals was almost identical at 2 weeks PC (**Figure 6C**). The MFTs response was further evaluated at 4 weeks PC; however, no statistically significant difference was found among experimental or control groups. Nevertheless, the level of induced CD4+ MFTs was still higher in PII treated animals compared to all other immunized groups (**Figures 6B, D**).

### DPP Based Immune-Modulation Scheme Ensues in Development of Effector as Well Central T Cell Memory Following Mtb Challenge

**Figure 7:**
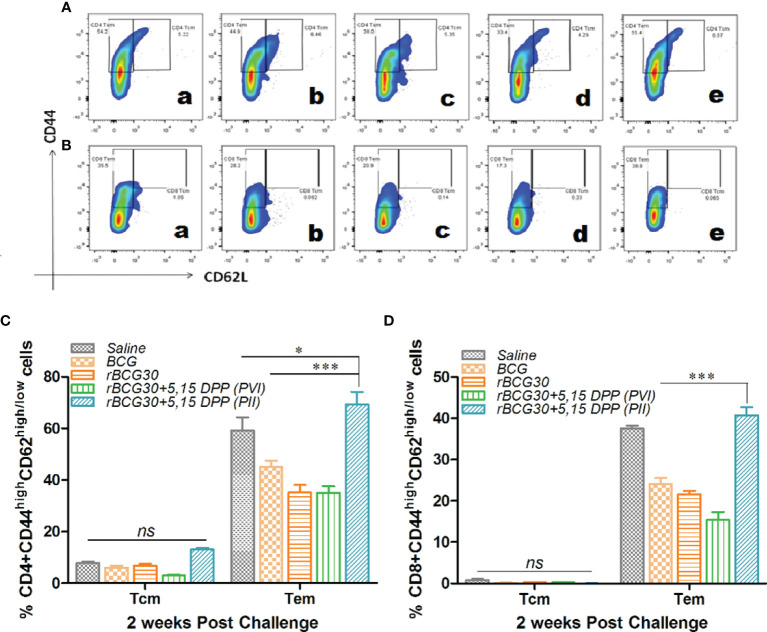
Memory T cell response at week 2 post-challenge. Antigen-specific CD4+ and CD8+ T central and effector memory response was assessed in the splenocytes isolated from various groups at 2 weeks PC. Shown in the figure are representative FACS plots depicting cumulative frequencies of **(A)** CD4+ and **(B)** CD8+ memory T cells from (a) Saline, (b) BCG, (c) rBCG30, (d) rBCG30+5,15-DPP (PVI), and (e) rBCG30+5,15-DPP (PII) groups, respectively. Bar graphs in the figure are depicting comparative magnitude (in percent) of effector memory (CD44^high^CD62L^low^) (Tem) and central memory (CD44^high^CD62L^high^) (Tcm) among **(C)** CD4+and **(D)** CD8+ T cells. The data were analyzed by employing Two-way ANOVA followed by Bonferroni’s multiple comparison test and are shown as means ( ± SEM) from one of the two independent experiments with similar observations performed in at least three biological replicates, where p<0.05(*), p<0.01(**), and p<0.001(***) were considered significant. ns, non-significant.

Splenocytes isolated from various experimental and control animals were evaluated at 2 week PC for the presence of central (CD44^high^CD62L^high^) as well as effector (CD44^high^CD62L^low^) memory phenotype on both CD4+ and CD8+ T cell population. As is evident from **Figure 7**, IL-10/STAT3 directed therapy PC with *Mtb* (PII) resulted in an early and significant expansion of effector memory T cells (Tem) in rBCG30 vaccinated mice. Animals in the rBCG30+PII group had a significantly higher percentage of CD4+ and CD8+ Tem cells than animals in either rBCG30 or rBCG30+PVI groups (p<0.001). However, at 2 weeks PC, the frequency of CD4+ central memory T cells (CD4+ Tcm) was not significantly altered among the groups. Nevertheless, the frequency of CD4+ Tcm was still higher in the rBCG30+PII animals as compared to other control groups (**Figure 7C**). At 2 weeks PC, the level of CD8+ Tcm cells was negligible and inseparable among various groups (**Figure 7D**).

### DPP Therapy Led to Sustained and Systemic Transition of T Cell Memory in rBCG30 Immunized Mice

**Figure 8:**
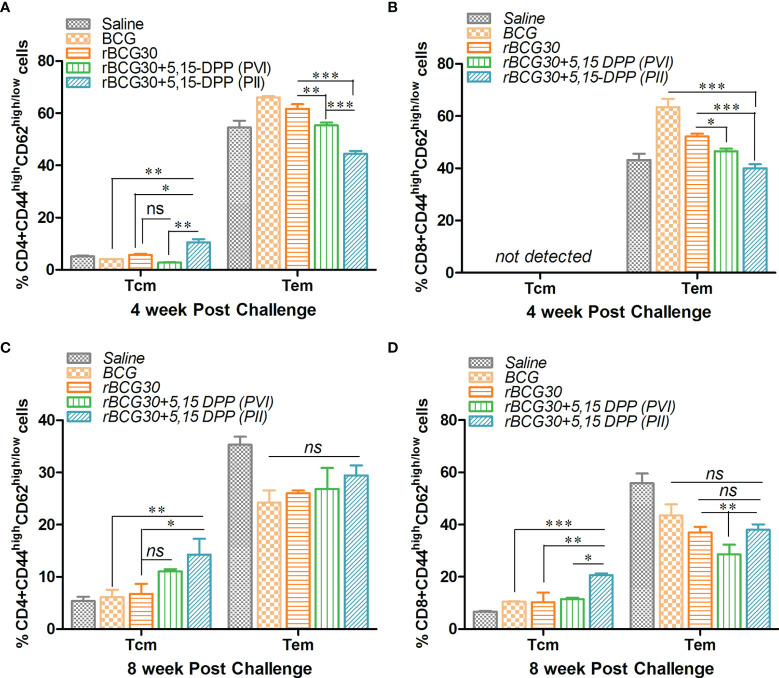
Evaluation of T cells memory response at 4 and 8 weeks post-challenge. Antigen-specific CD4+ and CD8+ T central and effector memory response was assessed in splenocytes isolated from various groups of mice at both 4 and 8 weeks PC. The bar graphs in the figure depict the comparative magnitude (in percent) of T effector (Tem) (CD44^high^CD62L^low^) and T central (Tcm) (CD44^high^CD62L^high^) cells among **(A, C)** CD4+, and **(B, D)** CD8+ memory T cells at 4 (upper panel) as well as 8 (lower panel) weeks PC, respectively. The data were analyzed by employing two-way ANOVA followed by Bonferroni’s multiple comparison test and are shown as means ( ± SEM) from one of the two independent experiments with similar observations performed in at least three biological replicates, where p<0.05(*), p<0.01(**), and p<0.001(***) were considered significant. ns, non-significant.

The effect of DPP based immunotherapeutic strategy on the maintenance of T cell memory was further evaluated at 4 and 8 weeks PC. The splenic T cells from immunized animals were examined for expression of established T cell memory markers CD44 and CD62L. Interestingly, in contrast to 2 weeks PC time point, both CD4+ and CD8+ short lived Tem cells were found to be significantly down-regulated at 4 weeks PC in rBCG30 immunized animals administered PII (p<0.001). The long lived CD4+ Tcm cells were augmented in the same rBCG30+PII group of animals at 4 weeks PC (p<0.05). The CD8+ Tcm cells remained undetectable at this time point (**Figures 8A, B**). Specifically, at 4 weeks PC, the frequency (cumulative) of CD4+ Tem cells was reduced to ≈40% (**Figure 8A**) from ≈70% at 2 weeks PC (**Figure 7A**). The level of CD8+ Tem cells at 4 weeks PC (**Figure 8B**) remained closely similar to its level at 2 weeks PC (**Figure 10B**). Interestingly, the level of CD4+ Tcm cells was increased in the rBCG30+PII group, as compared to either rBCG30 (p<0.05) or rBCG30+PVI (p<0.01) group (**Figure 8A**).

The decreasing trend of effector memory and augmentation of central memory T cells in the rBCG30+PII group was found to continue until 8 weeks PC with a significant drop in the cumulative level of short lived CD4+ Tem cells (from ≈44% to ≈29%) (**Figure 8**). Conversely, the level of CD4+ Tcm cells in the rBCG30+PII group remained high even at week 8 PC, as compared with either rBCG30 (p<0.05) or BCG (p<0.01) immunized groups (**Figure 8C**). Notably, the expansion of previously undetected CD8+ Tcm cells was spotted at week 8 PC. The CD8+ Tcm cells were induced significantly in rBCG30-immunized animals that received DPP therapy (PII) (p<0.001; **Figure 8D**). To our understanding, the significant induction of CD8+ Tcm cells along with CD4+ Tcm cells during the late phase of infection embodies the final combined push to resist the pathogen’s attempt to acquire dominance during the late phase of infection. Moreover, the failure of BCG is correlated with the predominant expansion of effector memory and weak (or no) central memory T cells. We observed predominance of Tem cells and few central memory cells early after infection. Nevertheless, in contrast to classical BCG or rBCG30 (control groups) based immunization, the level of both CD4+ and CD8+ Tcm cells at the later time point (week 8 PC) was high in the animals that received immunotherapy following aerosol *Mtb* infection (PII) that presumably helped prevent the establishment of chronic disease (**Figures 8C, D**).

### DPP Therapy Following Mtb Challenge Led to the Preferential Proliferation of Th17 Cells Over Immunosuppressive Tregs

**Figure 9:**
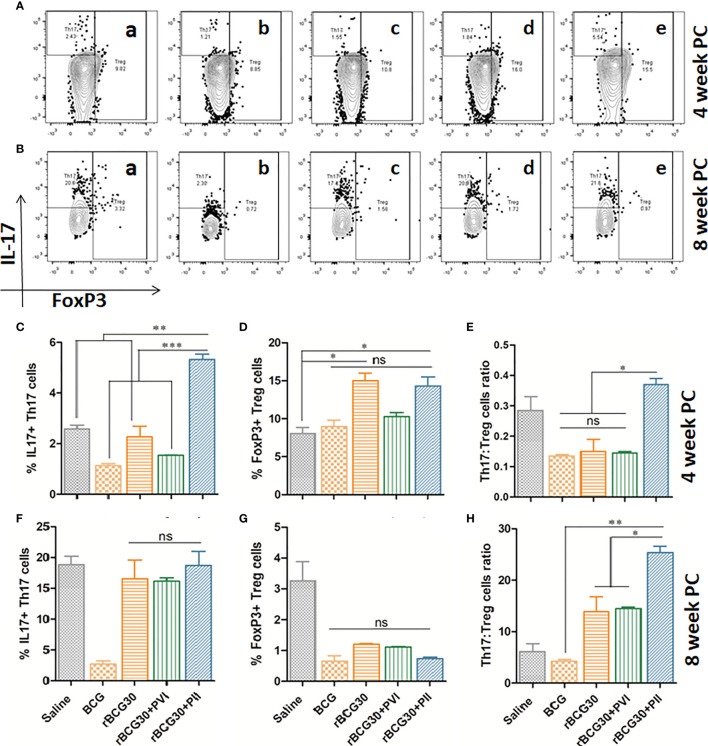
Th17/Treg cells dynamics in response to DPP based immunotherapy. Representative FACS micrographs in upper panel depicting Th17/Treg frequency at **(A)** 4 and **(B)** 8 weeks PC with groups labels **(a)** Saline, **(b)** BCG, **(c)** rBCG30, **(d)** rBCG30+5,15-DPP (PVI), and **(e)** rBCG30+5,15-DPP (PII), respectively. Bar graphs in the figure are depicting quantitative magnitude (in percent) of Th17 cells at weeks **(C)** 4 and **(F)** 8, Treg cells at weeks **(D)** 4 and **(G)** 8, and the ratio of Th17 to Tregs at **(E)** 4 and **(H)** 8 weeks PC. The level of significance of the data was tested using Two-way ANOVA followed by Tukey’s post-test and are shown as means ( ± SEM) from one of the two independent experiments with similar observations performed in at least three biological replicates, where p<0.05(*), p<0.01(**), and p<0.001(***) were considered significant. ns, non-significant.

To further establish the efficacy of DPP based immunomodulatory strategy targeting the immunosuppressive IL-10/STAT3 signaling axis; the comparative expression of CD4+ Th17 and Treg cells was assessed. The role of Th17 cells in providing protection from TB is much debated as they are considered dispensable for protection against *Mtb* (44). Nevertheless, the Th17 subset plays an adjunctive role in enhancing the diminished Th1 environment, consequent to the activity of anti-inflammatory mediators, including IL-10, during chronic *Mtb* infection (24). Immunosuppressive FoxP3+ T regulatory cells (Tregs) are known to inhibit effector anti-mycobacterial T cell responses and cause a delay in the onset of adaptive immunity (51–53). Thus, the magnitude of both Th17 and Treg cells was investigated. An attempt was made to establish the causal relationship between the two subsets by transforming their relative frequencies as a relative ratio (Th17:Treg) (**Figure 9**). While Th17 cells are known to back up deficient Th1 responses (24), Tregs are supposed to brake-off the proliferation of both Th1 and Th17 cells during mycobacterial infection (51, 53). We anticipate that the immunotherapeutic strategy directed towards fine-tuning of Th17/Treg counterbalance, predominantly towards Th17, may prove beneficial to achieve optimal protective immunity against TB.

In the present study, we observed improved Th17 cells proliferation over Tregs in the animals vaccinated with rBCG30 and those that received immunotherapy following *Mtb* challenge (PII) (**Figure 9**). At 4 week PC, animals belonging to the rBCG30+PII group displayed a significantly high Th17 to Treg ratio (p<0.05; **Figure 9E**), and the difference observed was even more at week 8 PC (p<0.001; **Figure 9H**). The observed heightened proliferation of IL-17 producing Th17 cells and relatively low frequencies of FoxP3+ Tregs reflect the ability of employed immunotherapeutic regimen to induce pro-inflammatory Th17 lymphocytes, while simultaneously limiting the differentiation of immunosuppressive Tregs.

### The Compound DPP Lacks Intrinsic Anti-Mycobacterial Activity Against Mtb Strain H37Rv *In Vitro*

In addition to immunomodulatory activity, one can argue that the DPP may possess direct anti-mycobacterial activity as well. To rule out the possibility of intrinsic anti-mycobacterial activity of the immunomodulator DPP, we performed *in vitro* antimycobacterial susceptibility testing employing 96 well microtiter plate based alamar blue assay on avirulent *Mtb* strain H37Ra as well as MGIT assay employing standard virulent *Mtb* strain H37Rv (**Figure S3**). The antibacterial assay data employing the above specified assays suggested that DPP does not possess intrinsic anti-mycobacterial activity. The DPP exhibited a level of antibacterial activity comparable to that of DMSO in inhibiting *Mtb H37Ra or Rv* outgrowth *in vitro* at the tested concentrations. The *in vitro* data ruled out any intrinsic anti-mycobacterial activity at the tested concentration.

### DPP Immunotherapy Following Mtb Infection Conferred Superior Protection in rBCG30 Vaccinated Mice

The *in vivo* efficacy of employed host-directed immunotherapy was assessed for its potential to augment the protective capacity of the rBCG30 vaccine against murine TB. The mice were immunized with rBCG30 and administered DPP based immunotherapy, either post vaccination or post-infection (with *Mtb*), were assessed for their ability to combat pulmonary *Mtb* infection. To evaluate the effect of immunotherapy on the control of mycobacterial infection *in vivo*, we examined mycobacterial loads in infected lungs and spleen from the various experimental and control groups of animals at 4 and 8 weeks PC.

**Figure 10:**
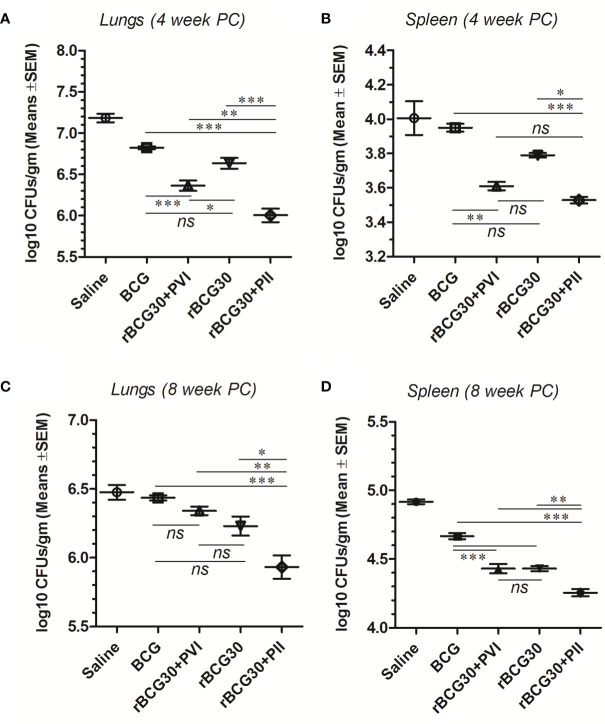
Residual *Mtb* burdens in the lungs and spleen of the infected mice. *Mtb* bacillary load was assessed in rBCG30-immunized and DPP treated (both PVI and PII setups) mice at week 4 and 8 post aerosol *Mtb* challenge. CFU loads in the organ homogenates were enumerated and expressed as log10 CFU/g of tissue. The scatter plots in the figure represent log10 CFU/g ± SD (of at least five biological replicates per group) in lungs and spleen at **(A, B)** 4; as well as **(C, D)** 8 weeks post-challenge, respectively. CFU data were analyzed by employing one-way ANOVA followed by Tukey’s post-test, where p< 0.05(*), p< 0.01(**), and p< 0.001(***) were considered significant. ns, non-significant.

Surprisingly, animals treated with DPP following aerosol challenge with *Mtb* (PII) displayed superiorly improved bacillary clearance in the lungs, when compared with either rBCG30 (p<0.001) or rBCG30+PVI groups (p<0.01). In spleen, the bacillary reduction in the rBCG30+PII group was significant in comparison with the rBCG30 group (p<0.05), but not with the rBCG30+PVI group (p>0.05). Moreover, in comparison with standard BCG control, organ bacillary reductions were prominent in PII treated animals (p<0.001), as well as the PVI treated animals (lungs, p<0.001; spleen, p<0.01), at 4 week PC (**Figures 10A, B**).

Bacillary burdens in the lungs and spleen of infected animals were further evaluated at week 8 PC. Similar to at 4 weeks PC time point, the CFU burden at week 8 PC was significantly suppressed in rBCG30-immunized animals that received PII with DPP. The adopted immunotherapeutic scheme was also able to reduce bacterial loads significantly at week 8 PC in rBCG30+PII treated animals, as compared to either classical BCG (p<0.001) or rBCG30 vaccinated controls (lungs, p<0.001; spleen, p<0.01), and/or post-vaccination immunotherapy (PVI) administered animals (p<0.01) (**Figure 10C, D**).

**Figure 11:**
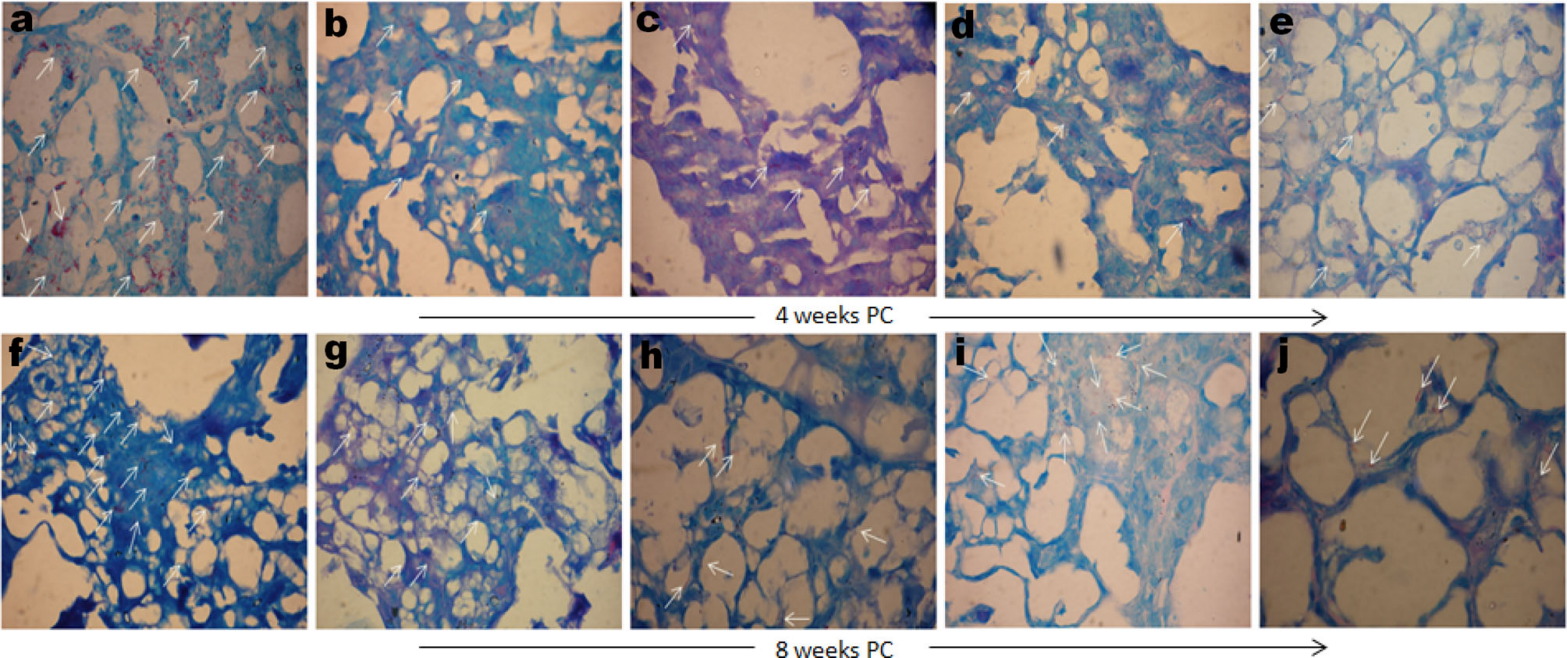
Gross tissue mycobacterial loads in the infected lungs. Representative photomicrographs showing differential *Mtb* bacillary loads in the lungs tissue sections of immunized and DPP immunotherapy administered animals at 4 (upper panel) and 8 (lower panel) weeks PC. Arrows indicating Ziehl–Neelsen dye stained *Mtb* bacilli in various immunized and treated groups (Original magnification—1000× in oil immersion lamp). The various groups were as follows: **(A, F)** Saline** (B, G)** BCG** (C, H)** rBCG30, **(D, I)** rBCG30+PVI, and **(E, J)** rBCG30+PII, respectively.

Comparative mycobacterial loads in the lungs of various immunized and treated groups of animals were also evaluated by AFB staining of the lungs tissue sections. Formalin-fixed lungs tissue sections were subjected to Ziehl–Neelsen staining to assess the comparative tissue bacillary loads in various immunized and treated mice at 4 as well as 8 weeks PC. Mice administered DPP therapy following *Mtb* challenge displayed significantly fewer numbers of AFB+ bacilli in lungs tissue sections stained with Ziehl–Neelsen dye at either 4 or 8 weeks PC, as compared to either saline, BCG, or rBCG30 administered animals (**Figure 11**). Additionally, rBCG30 immunized and PVI treated animals didn’t display improved bacillary clearance at either 4 (**Figure 11D**) or 8 (**Figure 11I**) weeks PC time points and the gross tissue mycobacterial loads were equivalent to that present in rBCG30 immunized control animals. Interestingly, while in all other groups bacilli were stained as multibacillary aggregates, in rBCG30+PII administered animals there were usually one or two bacilli at particular foci (paucibacillary), which suggest restrained *Mtb* growth in the group. In concordance to CFU enumeration data, lung tissue from PII treated animals displayed the least AFB+ burdens, as compared to rBCG30 alone or rBCG30+PVI treated mice at 4 as well as 8 weeks PC (**Figure 11**).

Overall, there was a maximum reduction in organ CFU burden in the animals vaccinated with rBCG30 and treated PC with DPP (PII). The *in vivo* protective efficacy data of the present study further underscore the protective potential of IL-10/STAT3 targeted DPP therapy (PII) in bolstering host resistance as well as the efficacy of rBCG30 vaccine against *Mtb* challenge in mice.

## Discussion

Host directed (immuno) therapeutic (HDT) strategies against *Mtb* have become a research focus in the eradication of TB disease (reviewed elsewhere) (54). Incidentally, most of the tested schemes were proposed either as stand-alone or as an adjunct to standard anti-TB chemotherapy. Despite this, efforts aimed towards using host immune machinery to convalesce the prophylactic immunity conferred by BCG or its engineered recombinant version are relatively limited (23, 53, 55). The IL-10/STAT3 signaling axis has been implicated in generating pro-angiogenic/anti-inflammatory responses in the host (5, 25, 26). Multiple reports have shown that lL-10 persuades STAT3 to skew *Mtb* infected monocytes/macrophages towards pathogen permissive, anti-inflammatory AAM state (3, 6, 31–33). IL-10 has been considered to be a potent pro-mycobacterial cytokine that opts to weaken/disable anti-mycobacterial immune responses (21, 56). Apart from its active role in the direct inhibition of Th1 cell expansion (57), IL-10 delays the migration of DCs from *Mtb* infected lungs to draining lymph nodes (22). Rather, the cytokine IL-10 rejuvenates the chronic/latent *Mtb* infection (20). An optimum vaccination strategy to counter *Mtb* could thrive if it induces a protective pro-inflammatory response in the host while simultaneously restraining pathogen-triggered anti-inflammatory or regulatory pathways. Bearing this in mind, the present study explored DPP based immunotherapy as a host targeted intervention to enhance rBCG30 vaccine mediated immunity against experimental murine TB.

The DPP based therapeutic modulation of host immunity resulted in the significant expansion of M1 monocytes/macrophages (CAMs) along with concomitant reductions in the numbers of M2 monocytes/macrophages (AAMs) in the peritoneum as well as the spleen of the PVI (**Figures S1, S2**) or PII (**Figures 1A–C**) administered animals; except a feeble expansion of classical macrophages in the spleen of the PII administered group (**Figure 1D**). The differential repertoire of peritoneal and splenic monocytes/macrophages in the PII group can be explained based on two propositions. First, intra-peritoneal administration of DPP therapy might have re-programmed the peritoneal monocytes/macrophages toward a classical state more promptly as compared to their splenic counterparts. Second, because aerogenic *Mtb* takes around two weeks to reach the splenic compartment from the aerosol infected lungs (22), the splenic resident cells in PII administered animals might not have been signaled to alter their steady state phenotype, as nearly 70-80% splenic monocytes/macrophages were found to be in the M2 state until then. By contrast, at 4 weeks PC, both classical monocytes and DCs were significantly expanded in the PVI group, macrophages from the same group of animals did not display any significant difference in numbers. Interestingly, animals belonging to the PII group showed increased numbers of monocytes, macrophages, and DCs with a classical phenotype at this point (**Figure 2**). This suggests that PII with DPP was still better at 4 weeks PC in maintaining a pro-inflammatory environment in macrophages, the primary cells *Mtb* thrives in, as opposed to PVI.

The phenotypic polarization status of the mononuclear phagocytes was further assessed at the week 8 PC time point. As the population of DCs was below the detection level; therefore, we confined our investigation to monocytes and macrophages only. It is noteworthy that the animals from the PVI group failed to maintain a pro-host innate immune response for long and instead displayed predominant differentiation of anti-inflammatory monocytes/macrophages (AAMs) at week 8 PC (**Figure 3**). The weak induction of macrophages bearing classical phenotypes during the late phase of *Mtb* infection can be correlated with the failure of the PVI strategy to execute bacterial clearance from the host. The maintenance of increased CAMs numbers in the PII group can have a direct correlation with the sustained and controlled expansion of pro-inflammatory milieu that might, in turn, ensued in superior protection of the immunized animals (**Figure 3**). As assessed from the mononuclear phagocytes dynamics, it can be inferred that the post vaccination immunotherapy (PVI), executed before infection with *Mtb*, may be of minimal benefit to the host. It could also be attributed to the fact that avirulent BCG strains are fundamentally different from virulent *Mtb* in terms of pathogenesis (58–60).

For its unhindered intracellular survival, *Mtb* skews invading monocytes/macrophages/DCs toward the M2 state and inhibits the secretion of Th1 cytokines (22, 61). Notably, we found up-regulation of Th1 cytokines (IL-12, IFN-γ) and down-regulation of hallmark Th2 cytokines (IL-10, IL-4) in DPP treated animals following the conclusion of both PVI or PII immunotherapy schedules (**Figure 4**). The level of Th1 cytokine IL-12 was found up-regulated even until 8 weeks PC in the PII group of animals, but not in the PVI group (**Figure 5A**). However, a transient dip in the level of another Th1 cytokine IFN-γ was observed at 4 weeks PC in both PII and PVI group of animals, which was dramatically regained later at week 8 PC (**Figure 5D**). This transient decline in the IFN-γ level was coincident with the significantly augmented level of IL-6 (**Figure 5B**) and IL-17+ Th17 cells expansion (**Figure 9C**) in PII administered animals. The observed cytokine modulation was in concordance with the previous report, where mycobacteria inhibited Th1 response was backed up by IL-23 (and possibly IL-6) mediated Th17 response, followed by restoration of functional Th1 response (24). The levels of innate cytokines IL-1β and IL-6 were not significantly affected in either PVI or PII treated animals until 2 weeks PC with *Mtb* (**Figure 4**). However, at 4 and 8 week PC, there was a significant increment in the level of IL-6 in PII administered animals (**Figure 5B**). The level of hallmark innate cytokine IL-1β was not significantly influenced during the course of infection (**Figure 5E**). Interestingly, IL-1β was least induced in rBCG30+PII administered animals at week 8 PC. The significantly reduced level of IL-β in the rBCG30+PII group may be considered as a correlate of the control of IL-β mediated exuberant inflammation during uncontrolled *Mtb* infection, as also reported previously (18). As expected, the DPP based immunotherapy transiently abrogated the expression of IL-10 until 4 weeks PC (**Figure 5F**), while IL-4 levels were indifferent in either of experimental and control groups at 4 or 8 weeks PC (**Figure 5C**), as opposed to significant inhibition observed at 2 weeks PC (**Figure 4D**). The observed transient reductions in the level of anti-inflammatory cytokines, especially IL-10, are in line with our efforts to fine-tune the anti-inflammatory signaling during the establishment of infection, instead of abrogating it permanently. The transient blockade of IL-10 signaling was desirable as it help avoid the inhibition of efficient immune priming (22). A complete absence of IL-10 increases the likelihood of developing pathological and auto-immune responses in the host. Consistently, these results implicate IL-10 as a primary culprit that limits vaccine-induced protective anti-mycobacterial immunity by modulating mononuclear phagocytes toward an alternative/anti-inflammatory state of activation. Taken together, the cytokines expression data concord to the previous proposition that the design of host directed therapies against TB must attain an optimal balance between pro- and anti-inflammatory signaling/responses, so as to induce the protective immunity in a manner that inflict minimal collateral tissue pathology (62).

Protection against intracellular pathogens including *Mtb* cannot be achieved without optimum stimulation of the T cell repertoire. In this regard, the multifunctionality of induced T cells upon vaccination or challenge with an infection is an important correlate to assess protective immunity (47). The enhanced proliferation of CD4+ MFTs at 2 weeks PC in the PII group of the animals (**Figure 6**) plausibly accentuated the immune-stimulatory profile and protective potential of the rBCG30 vaccine in immunotherapy (PII) administered animals. The induction of CD4+ MFTs has been considered as an important correlate of a candidate vaccination scheme to impart protective immunity against experimental TB (49, 63).

T cell memory response is an obligatory component of vaccine design to mediate impeccable protection against *Mtb* (64). The DPP immunotherapy mediated strategic manipulation of the IL-10/STAT3 anti-inflammatory axis led to the concerted proliferation of CD4+ and CD8+ effector (CD44^high^CD62^low^) and central (CD44^high^CD62^high^) memory T cells in the vaccinated host. The memory T cell compartment was initially (2 weeks PC) dominated by both CD4+ and CD8+ effector memory T cells (Tem) in the PII group (**Figure 7**), which later declined and was replaced by CD4+ and CD8+ central memory T cells (Tcm) subsets at week 4 and 8 PC (**Figure 8**). The frequency of Tcm (either CD4 or CD8) was heightened at week 8 PC in PII treated animals, with a significantly high level noted of the CD8+ Tcm population (**Figure 8**). It is well established that antigen exposed ‘epigenetically programmed’ effector T cells generally result in a subsequent burst of long lived memory T cells (65). Intriguingly, the observed ‘systemic transition of Tem population to Tcm’, observed in the present study is of particular interest and is in line with current dogma, which argues for differentiation of long lived Tcm cells from the pool of antigen experienced, memory precursor Tem cells, in an ordered, epigenetically regulated ‘On-Off-On’ process (65–67). The T cell memory response data of the present study concord to this fact, as there was an early preponderance of Tem, and late differentiation of Tcm cells that probably were expanded from among contraction phase survived Tem cells. The greater proportion of effector T cells surviving the contraction phase and entering into a long-lived memory state is indeed an outcome of DPP immunotherapy. This eventually led to unusual induction of T cells with central memory phenotype during the late phase of infection that actually might have restricted the establishment of chronic *Mtb* infection. The observed decline in short lived Tem cell population, specifically at later time points, underscores the relevance of the proposed immunotherapy strategy in restricting surplus effector responses. Notably, the classical BCG vaccine predominantly induces Tem cells and fails to evoke sufficient protective Tcm cells (68). Thus, the employed immunotherapy may also be explored concomitantly with chemotherapy for its potential to rejuvenate the host immune armory in BCG vaccinated subjects who present with clinical *Mtb* infection.

Fine-tuning between Th17 and Treg cells proliferation is another important aspect in effectively tackling *Mtb* infection (69, 70). In this regard, we measured the relative proliferation of Th17 and Treg cells in various immunized groups and employed the Th17/Treg ratio as an indicator to determine the protective capacity of the proposed immunotherapeutic strategy. We observed increased proliferation of Th17 cells with concomitant downregulation in the frequency of FoxP3+ Treg cells in the immunized animals under the PII scheme (**Figure 9**). While Th17 cells play an important role in protection against TB (23, 70), Tregs are known to dampen anti-TB immunity and contribute to the progression of the disease (51, 71). The increased Th17:Treg ratio, both at 4 and 8 weeks PC, further affirms the ability of DPP based immunotherapy to selectively inhibit the anti-inflammatory cues following *Mtb* infection in the vaccinated animals (**Figure 9**).

To assess the protective efficacy of the proposed immunotherapeutic strategy, an *in vivo* protection study was conducted in the mice. The prophylaxis data further affirm that the immunotherapy under the PII scheme resulted in improved protection against aerosol *Mtb* challenge. The immunotherapy (PII) was successful in reducing the bacillary loads in the lungs and spleen of infected mice up to weeks 8 PC, as compared with rBCG30-immunized and untreated control groups (**Figure 10**). Additionally, the results from histopathological studies (**Figure 11**) further establish the superior effectiveness of DPP therapy in reducing *Mtb* bacillary loads in the lungs of rBCG30 immunized animals. The mice immunized with rBCG30 and given DPP therapy following aerosol *Mtb* challenge (PII), significantly controlled lungs *Mtb* burdens, as compared to other control and treatment groups (**Figure 11**). The results corroborate the findings of the CFU counting assay and provide additional evidence of the superior efficacy of DPP mediated post-infection immunotherapy (PII) in rBCG30 immunized and *Mtb* challenged animals. We also evaluated whether DPP possesses any intrinsic anti-mycobacterial activity in an *in vitro* investigation conducted on the H_37_Ra as well the H_37_Rv strain of *Mtb* (**Figure S3**). The results from *in vitro* investigation ruled out any direct anti-mycobacterial activity of DPP and corroborate the central finding of this work that the DPP indeed targets the host and not the bacilli.

The augmented efficacy of the rBCG30 vaccine in the PII administered group of animals establishes the utility of host IL-10/STAT3 depletion (transient) as a viable means to improve TB prophylaxis. It is worth re-mentioning that the successful control of TB relies primarily on the host immune system as the majority of TB infected subjects (~90%) never develop active disease. Emerging evidence suggests that the smart manipulation of the host immune system during vaccination or infection can improve the level of host immunity provided by BCG (53, 55) and BCG based recombinant anti-TB vaccines.

The role of the IL-10/STAT3 signaling axis has been subjected to intense investigations lately and is suggested to have an active role in establishing pro-mycobacterial immune ambiance in the infected host (3, 6, 29, 31–33). SOCS3 and STAT3 are reported as two major regulators during TB infection (5). Mycobacteria possess the ability to impair the SOCS3 feedback loop that limits the IL-6 mediated pro-inflammatory environment. The ensuing IL-10 cytokine induces prolonged activation of STAT3 that in turn promotes an anti-inflammatory environment in the host (5). STAT3 had been associated with numerous mycobacterial survival strategies including blockade of phago-lysosome maturation, dampening of reactive oxygen and nitrogen species production, inhibition of autophagy and apoptosis, feeble secretion of Th1 cytokines, reduced presentation of antigens, and eclipsing of co-stimulatory molecules on APCs (5). Few earlier reports have also demonstrated the potential of IL-10 inhibition during vaccination as an effective strategy to enhance anti-mycobacterial immunity (19, 23). Moreover, few recent reports emphasize the role of STAT3 in promoting *Mtb* infection in human subjects (33) and surrogate models (3, 6).

To the best of our knowledge, the present report is the first such attempt to use a small molecule, DPP, that inhibits IL-10 induced STAT3 dimerization and nuclear translocation by binding to either SH2 (Src homology 2) domain (37) or *via* interaction with cytosolic thioredoxin oxidoreductase (TrxR1) (72, 73). Enzyme TrxR1 has recently been described as a direct target of perceived STAT3 inhibitors (including DPP) (72, 73). The majority of available STAT3 inhibitors have been reported to directly bind with cytosolic oxidoreductase TrxR1 *in vitro* in a concentration dependent manner (73). The reduction of oxidized STAT3 by TrxR1 has been proposed to induce dimerization and nuclear translocation of STAT3 (72). It has been speculated that the majority of STAT3 inhibitors work indirectly rather than direct binding to STAT3 (72, 73). In the present study, we made use of the DPP as an immunomodulator/adjuvant, which also targets enzyme TrxR1 to down-modulate STAT3 exerted anti-inflammatory effects. Findings by Busker et al. (72, 73) and Mtwebana et al. (74) indicate that DPP may not directly bind to STAT3 and the observed effects might be STAT3 activation/phosphorylation independent, as also reported by Uehara and colleagues (37) and O’Farrell et al. (25). However, it is unlikely that they operate independently of the transcriptional activity of STAT3, which is extensively established to have a key role in generating an anti-inflammatory environment conducive for infection or cancer outgrowth.

Though this study does not provide direct evidence regarding STAT3 activation/inhibition, the presented evidence from cellular/immunological and protection experiments could be considered a direct correlate of DPP’s ability to tame anti-inflammatory effectors, most likely *via* its action on STAT3. We aim to discern the precise role and mechanism of DPP in providing protection against TB in further studies, which will seek to address the critical mechanistic gaps of the present study.

The present study establishes the potential of intercepting host IL-10/STAT3 (and possibly TrxR1) signaling as a crucial means to bolster innate immune defenses and subsequent adaptive immunity during *Mtb* infection. The proposed strategy is a proof-of-concept that highlights the exploration of IL-10/STAT3/TrxR1 directed immunotherapy as a promising approach to enhance vaccine immunity against TB. Moreover, the chemical nature of the immunomodulator DPP has great advantages in terms of cost-effectiveness, ease of production, stability, and delivery issues, as compared to antibodies or other bio-therapeutics based approaches employed in the earlier studies. The proposed immunotherapeutic approach can be exploited as a viable strategy in improving the protective profile of existing and forthcoming TB vaccines. In addition, it would also be equally interesting to evaluate if the DPP in conjunction with standard anti-TB chemotherapy may serve to enhance the chemotherapeutic index of standard anti-TB drug regimens.

## Materials and Methods

### Reagents

All standard reagents used in the study were purchased from Sigma-Aldrich (St. Louis, MO, USA) unless otherwise noted. The bacterial culture reagents, Middlebrook 7H9 broth; Middlebrook 7H11 agar, and Oleic Acid Albumin Dextrose and Catalase (OADC) supplement were procured from Difco Laboratories (Sparks, MD, USA). Plasticware was purchased from BD Biosciences (San Jose, CA, USA).

### Animals

6-8 weeks old, BALB/c mice (n=111) of male sex were used in the study. Animals were acclimatized for a week before commencing the experiments. All animals were kept in a resting state with free access to food and water for 2 months from the day of vaccination, except the post vaccination immunotherapy (PVI) group that was administered DPP daily for one week after vaccination with rBCG30.

### Immunization and Immunotherapy Schedule

Mice were immunized subcutaneously with either commercial BCG vaccine manufactured by Serum Institute of India or in-house propagated rBCG30-ARMF-II^®^ Tice. Vaccines were equilibrated to deliver 1-1.5×10^5^ CFUs in 100 µl of normal saline and administered subcutaneously. As a placebo, normal saline was administered in one group of animals (saline control group). Animals immunized with rBCG30 were divided into two sub-groups. The first group received post vaccination immunotherapy (PVI) with DPP (Sigma-Aldrich) daily for one week commencing at day 1 post vaccination. The second group received post-infection immunotherapy (PII) on alternate days for two weeks starting at day 1 post aerosol *Mtb* challenge. To negate the effect of DMSO used as a solvent and vehicle to deliver DPP, DMSO sham control groups, rBCG30+sham (PVI) and rBCG30+sham (PII), were also included in the study. No specific effect was observed on the DMSO carrier that was used as a solvent to deliver DPP. All the treatment with DPP or vehicle (DMSO) was given intra-peritoneum. The dose used of DPP was 15 mg/kg of body weight as described previously (26), in injection volume of 200 ul/mice/day.

### *Mycobacterium Bovis* BCG, rBCG30, and *Mycobacterium Tuberculosis* H_37_Rv Strains

rBCG30-ARMF-II^®^ Tice was provided by Professor Marcus A. Horwitz (UCLA) and obtained under the limited, non-commercial use agreement between UCLA (USA) and AMU (India). The Bacille Calmette–Guerin (Russian strain) was obtained commercially (marketed as Tubervac^®^ by Serum Institute of India) *Mtb* H_37_Rv strain was kindly provided by The Director, National JALMA Institute for Leprosy and Other Mycobacterial Diseases (NJIL and OMD), Agra, India. *Mtb* was cultured in Middlebrook 7H9 broth containing 0.2% glycerol and 0.05% Tween-80 supplemented with 10% OADC at 37°C as a shaking culture. rBCG30 was cultured in Middlebrook 7H9 broth containing 0.02% Tween-80 supplemented with 10% OADC at 37°C with continuous shaking. The *Mtb* H_37_Rv used in the study was passaged in mice on regular basis to ascertain its virulence. The viability was determined by culturing the bacteria on Middlebrook 7H11 medium supplemented with OADC and counting the number of colony forming units (CFUs).

### Antigens for *Ex Vivo* Stimulation of Immune Cells

Purified native Ag85B (5 μg/ml) was used to stimulate the cell cultures derived from various experimental and controls groups. Purified native Ag85B (NR-14857) was procured through BEI Resources (Manassas, VA, USA) under a TB research material procurement contract between NIAID (USA) and AMU (India). As per the manufacturer, the protein was purified from the culture filtrate proteins of *Mycobacterium tuberculosis* (strain H_37_Rv).

### Antibody Based Reagents and Assay Kits

The following fluorochrome-labeled anti-mouse antibodies were procured from e-Biosciences and BD Biosciences: anti CD3 (145-2C11), anti CD4 (GK 1.5), anti CD8 (53-6.7), anti CD44 (IM7), anti CD62L (MEL-14), anti CD11b (M1/70), anti F4/80 (T45-2342), anti Ly6C (AL-21), anti TNF-α (MP6-XT22), and anti IFN-γ (XMG1.2). Mouse Th17/Treg phenotyping kit (Cat. No. 560767) and IL-1β, IL-4, IL-6, IFN-γ, IL-12, IL-10 BD OptEIA cytokine ELISA kits were procured from BD Biosciences (USA).

### Ethics Statement

All animal experiments were approved by the Institutional Animal Ethics Committees (IAEC) of the ICMR-National JALMA Institute for Leprosy and Other Mycobacterial Diseases, Agra, India. All animal experiments were performed according to the National Regulatory Guidelines issued by CPCSEA.

### Establishment of Infection and Determination of Tissue Mycobacterial Loads

Two months post vaccination; all animals were challenged with virulent *Mtb* (H_37_Rv) through aerosol route. A bacterial suspension corresponding to 5 × 10^7^ bacteria/ml in 10 ml normal saline was added to the nebulizer unit of the Aerosol Inhalation Exposure System (Glas-Col, USA). To determine the number of viable bacilli delivered to and surviving in mice lungs, 3 animals were euthanized within 16 h post-challenge (Day 1), and the entire lung homogenates were plated onto 7H11 agar plates to approximate the average implanted CFUs in the lungs of infected animals. On average, ≈110 viable bacilli were deposited into the lungs of each mouse. All animal challenge/infection studies were performed in the BSL-3 level containment facility at ICMR-National JALMA Institute for Leprosy and Other Mycobacterial Diseases, Agra, India. Standard biosecurity and institutional safety procedures have been adhered to as per institutional SOPs and guidelines.

To evaluate the protective efficacy of the employed immunotherapeutic strategy, we determined the bacterial load in the lungs and spleen of experimental animals at 4 and 8 weeks post *Mtb* aerosol challenge. At stipulated time intervals, a minimum of 4 animals from each group were euthanized; their spleen and lungs were removed aseptically and homogenized in 7H9 media. Four different dilutions of prepared homogenate were plated onto 7H11 agar plates supplemented with OADC. Thiophene carboxylic acid hydrazide (TCH) at a concentration of 2 mg/ml was added to inhibit the growth of BCG or rBCG30in immunized groups. All the plates were incubated for 3–4 weeks at 37°C in a CO_2_ incubator with a constant supply of 5% CO_2_. After incubation, colonies were counted to calculate the bacterial load. Bacterial loads were interpreted and expressed as mean log_10_ CFU/g in the lungs and spleen of infected animals.

### Isolation of Lymphocytes, Mononuclear Phagocytes, and Peritoneal Exudates Cells

Mice from various experimental groups (*n* = 3-5) were euthanized at specific time points, i.e. 2 weeks (next day after the conclusion of post-infection immunotherapy (PII), 4 weeks, and 8 weeks post-challenge (PC) to assess immunological parameters in the immunized animals. Single cell suspensions of the spleens were prepared according to previously reported procedures (75). Briefly, spleens from animals representing various groups were macerated using frosted glass slides and passed through a 70 μM cell strainer to obtain single cell suspensions. The cell suspension was treated with ACK lysis buffer to lyse erythrocytes. Next, the cells were washed with Hanks Balanced Salt Solution (HBSS) three times and re-suspended in a complete RPMI 1640 medium. Peritoneal macrophages or Peritoneal Exudates Cells (PECs) were also isolated from PVI or PII treated animals the day after the completion of DPP therapy to assess their activation state specific phenotype. Isolated lymphocytes and mononuclear phagocytes from the spleen or peritoneum were characterized by assessing the presence of specific cell surface markers employing flow cytometry.

### Cytokine Assay: Assessment of Antigen Induced Cytokine Profile

Both pro (IFN-γ, IL-12, IL-1β, IL-6) and anti (IL-4, IL-10) inflammatory cytokines induced in *ex vivo* re-stimulated splenocyte culture supernatants (from various experimental and control groups) were estimated using OptEIA sandwich ELISA kits (BD Biosciences). Briefly, 100 μl of the purified capture antibodies were adsorbed overnight on polystyrene micro-titer plates (Maxisorp, Thermo Scientific) at 4°C in the kit recommended coating buffer. Plates were washed five times with PBST and blocked with 1% BSA. After washing, 100 μl of the supernatant (isolated from cultured splenocytes after 24 h) was dispensed in each well. After incubation for the time stipulated, the plates were thoroughly washed and incubated with respective biotinylated anti-mouse detection antibody. Afterward, the plates were washed three times with PBST. Subsequently, 100 μl of streptavidin-HRP conjugate was added to each well, and the plate was incubated for 30 min at room temperature (RT). The plates were again washed three times with PBST and finally a colored complex was developed with tetra methyl benzidine (TMB). The absorbance was read at 450 nm with a micro-titer ELISA plate reader (Bio-Rad).

### Flow Cytometric Phenotyping of T Lymphocytes and Mononuclear Phagocytes

*Ex vivo* re-stimulated splenic lymphocytes/phagocytes or PECs (unstimulated) were harvested and stained for flow cytometric analysis following a protocol provided by BD Biosciences. Briefly, 1×10^6^ splenocytes were washed twice with FACS staining buffer (PBS with 1% BSA and 0.1% sodium azide). Cells were incubated with Fc block (2.4G2) or with appropriate fluorochrome tagged monoclonal antibodies against CD3, CD4, CD8, CD44, CD62L, CD11b, F4/80, and Ly6C for 30 minutes at 4°C. After washing, cells were fixed with 4.0% paraformaldehyde (PFA). The flow cytometry data were acquired using FACS Aria-II platform with FACS Diva software (BD Biosciences) and a minimum of 10 000 events were recorded for each sample. Data were further analyzed with FlowJo software (Treestar Inc., USA). The cells of a definite phenotype [CD4^+^CD44^high^CD62L^low^/^high^ (CD4 Tmem), CD8^+^CD44^high^CD62L^low^/^high^ (CD8 Tmem) CD11b^+^F4/80^+^SSC^low^Ly6C^low^/^high^ (Macrophages), CD11b^+^F4/80^-^SSS^low^Ly6C^low^/^high^ (Monocytes), CD11b^-^F4/80^-^SSS^low^Ly6C^low^/^high^ (DCs)] were deduced as percentage of the gated cell population, as determined by flow cytometry.

### Intracellular Cytokine Staining for Multifunctional CD4+ and Th17/Treg Cells Detection

Splenocytes isolated and stimulated *ex vivo* for 24 h from various immunized mice were collected, washed with PBS, and stained for surface markers *viz.* CD3, CD4, and CD8 followed by fixation using Cytofix buffer (BD Biosciences). Thereafter, cells were permeabilized with Perm/Wash buffer (BD Biosciences), followed by intracellular staining to probe IFN-γ and TNF-α in the examined cells. GolgiStop solution (BD Biosciences) was added for the last 4 h of incubation before collecting the cells for staining and was removed subsequently through washing. Stained cells were subsequently acquired. The relative proportions of CD4+ Th17 and Treg cells populations were determined with a Th17/Treg phenotyping kit following the manufacturer-supplied protocol (BD). The stained cells were subsequently acquired on the FACS Aria-II platform with FACS Diva software (BD) and later analyzed by FlowJo software. For intracellular staining, a minimum of 50,000 events were recorded.

### Acid Fast Staining of Lungs Tissues

The experimental animals were sacrificed and their lungs were perfused fixed in 10% buffered formalin. Later, tissue blocks (of 3 mm × 5 mm dimensions) were processed for paraffin embedding, and subsequently, 10-mm thick sections were cut with a rotary microtome. Sections were subjected to Ziehl–Neelsen staining to identify and estimate the relative load of the acid-fast bacilli (AFB) in the stained tissue sections. Stained representative tissue sections were observed under a light microscope (Nikon). Observations were recorded and interpreted independently by an experienced histopathologist. Photomicrographs were taken from granulomatous regions of samples showing AFB+ staining.

### Microplate Alamar Blue Assay to Evaluate the Anti-Mycobacterial Activity of DPP

The direct anti-mycobacterial activity of DPP was evaluated following a previously published protocol with slight modifications (76). The avirulent H37Ra strain of *Mtb* (as a surrogate to check anti-*Mtb* activity in alamar blue assay) was grown in 100 ml of Middlebrook 7H9 broth (Difco) supplemented with 0.2% glycerol (Sigma), 10% OADC (Difco), and 0.05% Tween 80 (Sigma) on continuous shaking (180 rpm) at 37°C until the culture reached an optical density of 0.5 to 0.6 at 600 nm. The obtained bacterial suspension was washed with normal saline and suspended in 7H9 medium with OD adjusted to 0.01. The culture was then used for inoculation in 96 well microplate alamar blue assay in 100 ul 7H9 media (in duplicate). The compound DPP (dissolved in DMSO) was dispensed in each well at a two-fold diluting concentration (250 ug/ml to 1.95 ug/ml). The concentration of DMSO was maintained in all the wells and DMSO alone control was also included. Outer perimeter wells of the plate were filled with sterile water to prevent dehydration in experimental wells and the plate was sealed with parafilm. Medium alone was taken as a negative control and bacteria only as a positive control. Plates were incubated at 37°C for 5 days. On the fifth day post incubation in dark at 37°C, 50 µl of 0.3% alamar blue solution was added to all the wells, and the plate was further incubated for another 6 hrs. Post incubation, the absorbance of the colored complex was recorded at 570 nm (in absorbance mode) using a microplate spectrophotometric reader with wavelength correction at 600 nm (Eon, BioTek Instruments).

### Evaluation of the Direct Anti-Mtb Activity of DPP Using BACTEC MGIT960 System

We also determined the direct anti-*Mtb* activity of DPP *in vitro* using BACTEC mycobacterial growth indicator tubes (MGIT) 960 system (Cat. No. 445870-BD Biosciences). The MGIT960 system utilizes fluorescence readout as an indicator of mycobacterial growth. The MGIT tubes contained a fluorescent compound embedded in silicone on the bottom of the tubes. The fluorescent compound was sensitive to the presence of oxygen dissolved in the broth. Initially, the large amount of dissolved oxygen quenches emissions from the compound and little fluorescence can be detected. As the mycobacterial cells grow, they utilize dissolved oxygen and thus allow the dequenching of the fluorescent probe. The machine had a provision to automatically indicate when there is sufficient Mtb growth or at 42 days (cut-off if no growth). Upon unloading the tube from the machine, a trail gets generated for each unloaded tube that contains arbitrary fluorescent units. This enabled us to better understand the approximate growth of the culture.

The MGIT960 grown culture of *Mtb* H37Rv (approx. 10^5^-10^6^ CFU/ml) was used to perform the experiment. The freshly grown culture was diluted 100 times (GC100) and 100 µl of the diluted culture was added to 7 ml BBL MGIT tubes (Cat. No. 245122-BD Biosciences) supplemented with OADC, but no antibiotics were added. The DPP was added (in duplicate) at 50 µM concentration (100 µl volume) in tubes inoculated with GC100 and allow to grow in the system. In a negative control tube, 100 µl DMSO alone was added. Positive control (only bacteria) tubes were also included. The graph of fluorescence readout (arbitrary units) was plotted for experimental and control tubes.

### Statistical Analysis of Data

The data of various immunological studies (pertaining to various immunized and immunotherapy treated groups) was compared by employing either two-way or one-way ANOVA (as appropriate) followed by Bonferroni’s or Tukey’s multiple comparison post-test (as appropriate) using Graph Pad Prism software version 5.03. Data presented in various immunological assays are representative of at least 3 animals from each group and 2 similar experiments. The *p* values, <0.05(*), <0.01(**), <0.001(***) were considered as significant for analysis and interpretation of experimental data.

## Data Availability Statement

The original contributions presented in the study are included in the article/**Supplementary Material**. Further inquiries can be directed to the corresponding author.

## Ethics Statement

The animal study was reviewed and approved by Institutional Animal Ethics Committees (IAEC) of ICMR-National JALMA Institute for Leprosy and Other Mycobacterial Diseases, Agra, India. All animal experiments were performed according to the National Regulatory Guidelines issued by CPCSEA.

## Author Contributions

FA conceived the idea of the manuscript, carried out all major experiments, analyse and interpret most of the data and wrote the first draft of the manuscript. MO provided overall supervision of the study, analyzed the data, edited the manuscript critically, and acquired/provided funding for the completion of the study. PG and UG helped executed animal challenge studies and contributed reagents and resources. MSU, NK, and FJ helped carry out experimental investigations. SZ provided software resources and data analysis services. All authors contributed to the article and approved the submitted version.

## Funding

FA acknowledges University Grants Commission (UGC), India for granting him a Basic Science Research (BSR) fellowship for doctoral studies. No specific funding was acquired or used for conducting the study except fellowship/salary support to FA. F. No.25-1/2014-15(BSR)/7-6/2007(BSR), dated: 13 March & 24 June, 2015, against fellowship support to FA from UGC, India.

## Conflict of Interest

The authors declare that the research was conducted in the absence of any commercial or financial relationships that could be construed as a potential conflict of interest.

## Publisher’s Note

All claims expressed in this article are solely those of the authors and do not necessarily represent those of their affiliated organizations, or those of the publisher, the editors and the reviewers. Any product that may be evaluated in this article, or claim that may be made by its manufacturer, is not guaranteed or endorsed by the publisher.
