# Supplementary material for: Immunotherapy With 5, 15-DPP Mediates Macrophage M1 Polarization and Modulates Subsequent Mycobacterium tuberculosis Infectivity in rBCG30 Immunized Mice
Source: Front Immunol. 2021 Oct 29;12:706727. doi: 10.3389/fimmu.2021.706727 (PMC8586420; doi:10.3389/fimmu.2021.706727)
Supplement: Supplementary file 1 [file DataSheet_1.pdf]

Supplementary Information

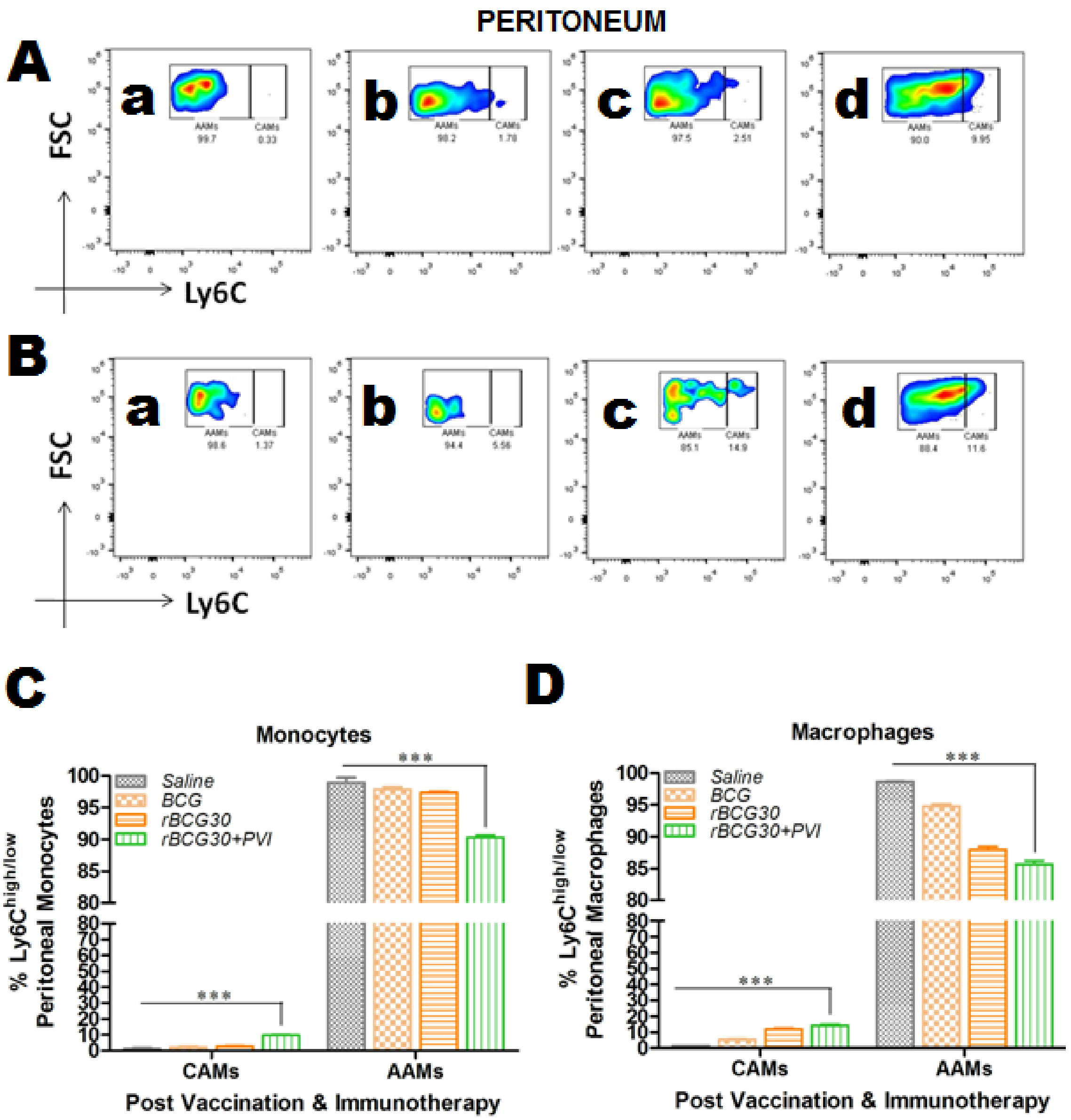

Fig.S.1. Peritoneal monocyte/macrophage phenotype in response to post vaccination immunotherapy (PVI). Representative FACS plots depicting cumulative frequencies of Ly6C^{low}AAMs and Ly6C^{high} CAMs among **(A)** CD11b^{+}F4/80^{-}SSC^{low} peritoneal monocytes, and **(B)** CD11b^{+}F4/80^{+}SSC^{low}peritoneal macrophages belonging to **(a)** Saline, **(b)** BCG, **(c)** rBCG30, and **(d)** rBCG30+5,15-DPP (PVI) groups, respectively. Bar graphs in the figure represent percent abundance of Ly6C^{high} CAMs and Ly6C^{low} AAMs among **(C)** monocytes, and **(D)** macrophages, isolated from peritoneal cavity of mice belonging to various immunized groups. The results depicted in the bar graphs are representative of two independent experiments and are presented as means ±SEM. The significance testing of variance among various groups was performed employing Two-way ANOVA followed by Bonferroni's multiple comparison post-test. The *p*

values, <0.05(*), <0.01(**), <0.001(***), were considered as significant for analysis of experimental data.

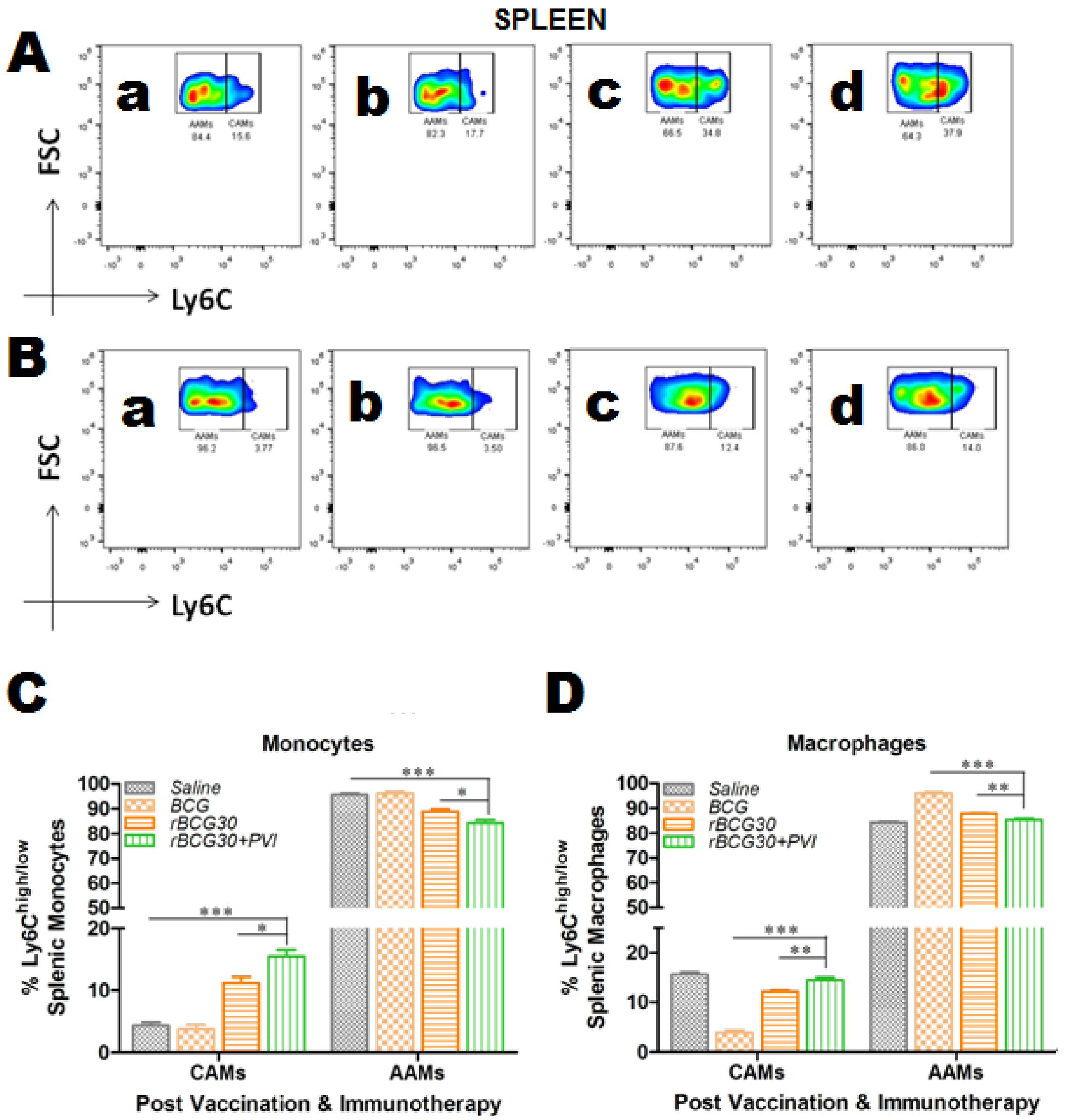

Fig.S.2. Splenic monocyte/macrophage profile following post vaccination immunotherapy (PVI). Representative FACS plots in the figure are depicting cumulative frequencies of Ly6C^{low} AAMs and Ly6C^{high} CAMs among **(A)** CD11b^{+}F4/80^{-}SSC^{low} splenic monocytes, and **(B)** CD11b^{+}F4/80^{+}SSC^{low}splenic macrophages belonging to **(a)** Saline, **(b)** BCG, **(c)** rBCG30, and **(d)** rBCG30+5,15-DPP (PVI) groups, respectively. Bar graphs represent percent abundance of Ly6C^{high} CAMs and Ly6C^{low} AAMs among (**C)** monocytes, and **(D)** macrophages, isolated from spleens of mice representing various immunized groups. The results depicted in the bar graphs are representative of two independent experiments and are presented as means ±SEM. The

statistical significance between various groups was tested employing Two-way ANOVA followed by Bonferroni's multiple comparison post-test. The *p* values, <0.05(*), <0.01(**), <0.001(***) were considered as significant for analysis and interpretation of data.

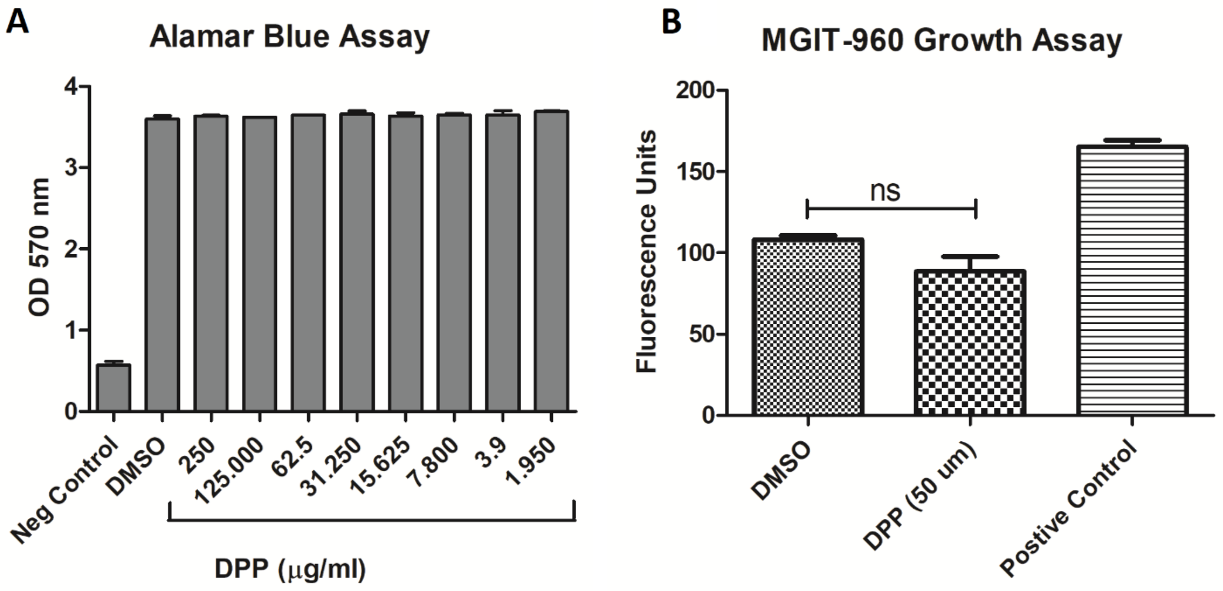

Fig.S.3. DPP displayed no direct anti-mycobacterial activity. The figure shows *Mtb* H37Ra **(A);** or H37Rv (**B),** growth in presence of various concentrations of DPP. No direct *in vitro* inhibitory activity was found of DPP against either *Mtb* H37Ra or H37Rv**.**

FACS GATING STRATEGIES

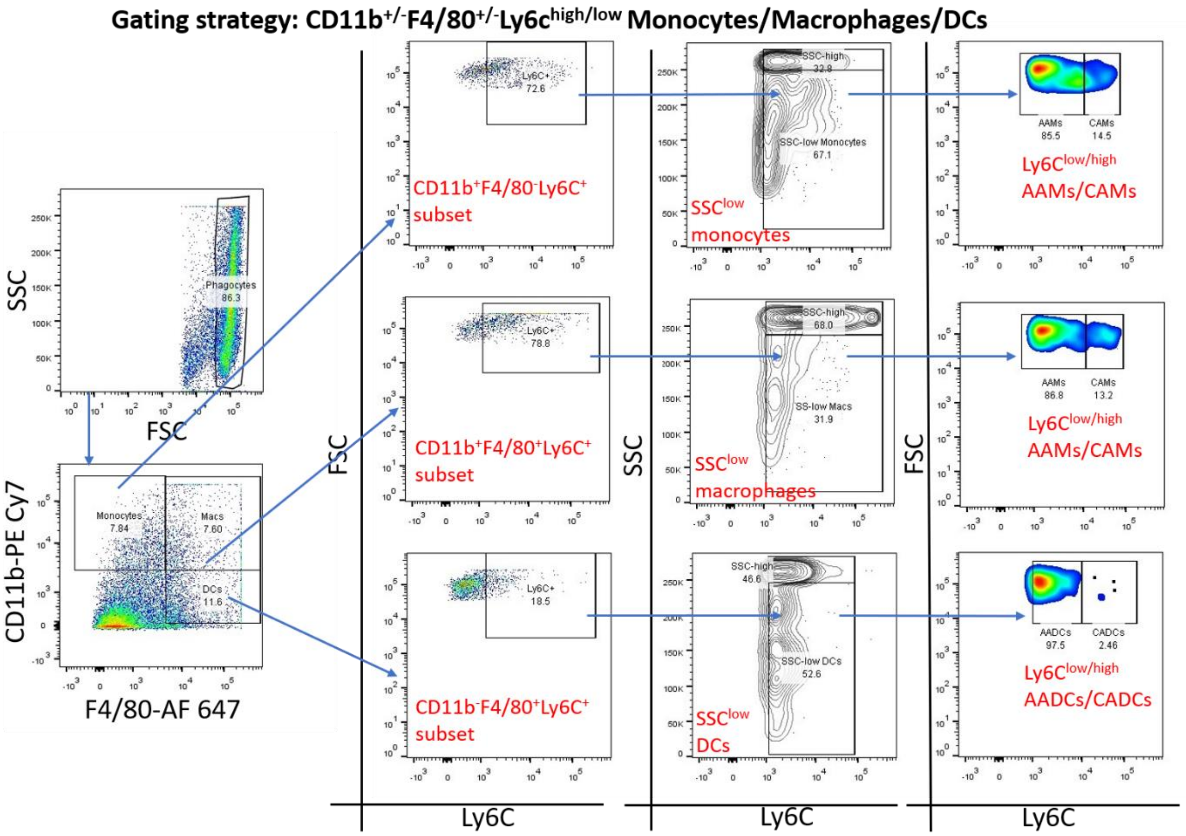

Fig.S.4. Gating strategy to determine CAMs/CADCs and AAMs/AADCs in spleen/peritoneum.

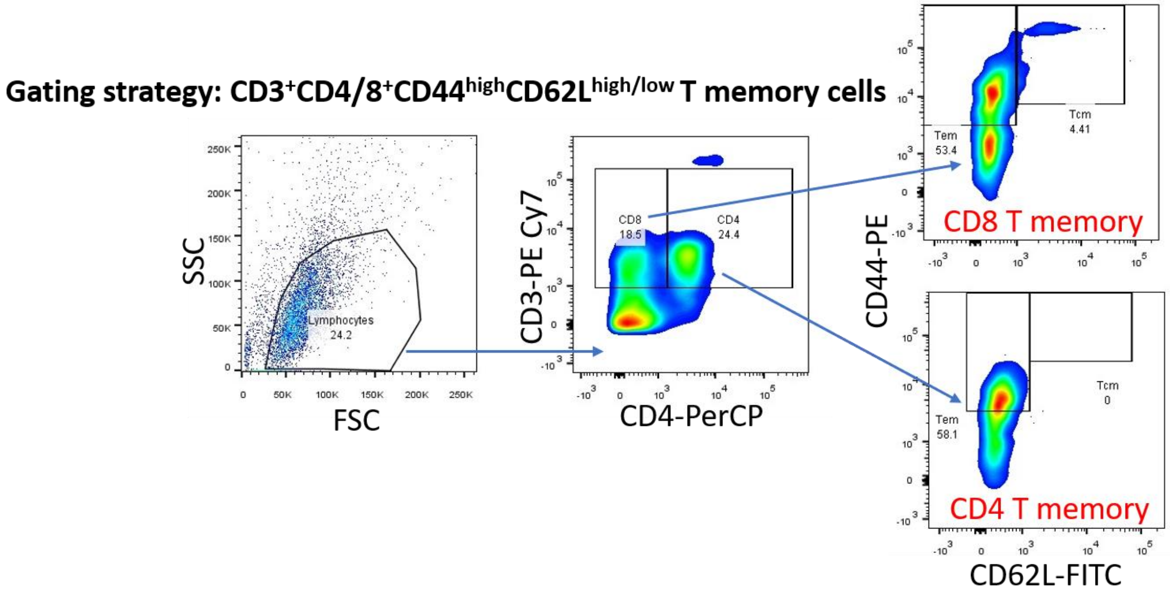

Fig.S.5. Gating strategy pertaining to memory T cells determination in splenocytes.

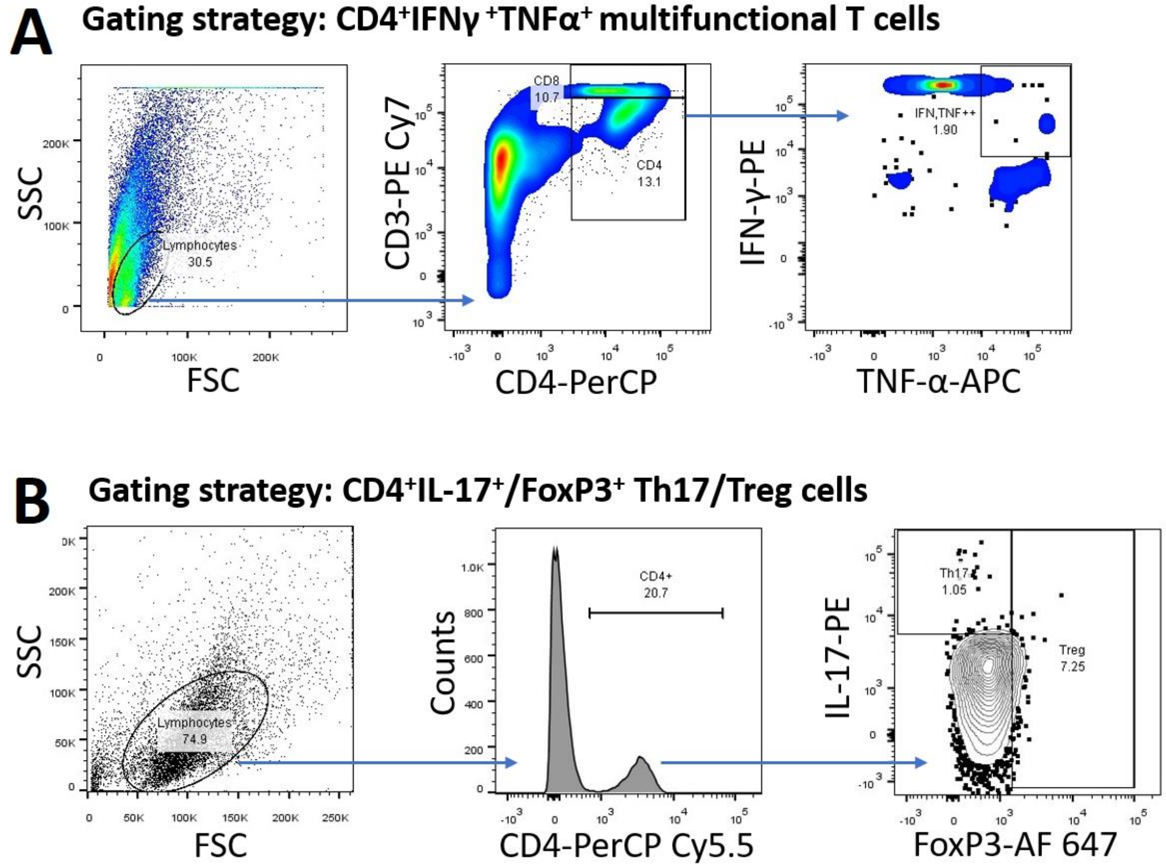

Fig.S.6. Gating strategy to determine (A) CD4+ multifunctional T cells and, (B) Th17/Treg cells.
